# Beyond Hemorrhagic Fever: Hepatic and Endothelial Dysfunction in Ebola Virus Disease

**DOI:** 10.3390/biology15201798

**Published:** 2026-10-09

**Authors:** Francesco De Maria, Francesco Branda, Giancarlo Ceccarelli, Fabio Scarpa, Massimo Ciccozzi, Alessandro Russo

**Affiliations:** 1 Infectious and Tropical Diseases Unit, “Renato Dulbecco” Teaching Hospital of Catanzaro, 88100 Catanzaro, Italy; francescodemaria16@gmail.com (F.D.M.); a.russo@unicz.it (A.R.); 2 Unit of Medical Statistics and Molecular Epidemiology, Università Campus Bio-Medico di Roma, 00128 Rome, Italy; m.ciccozzi@unicampus.it; 3 Department of Public Health and Infectious Diseases, University Hospital Policlinico Umberto I, Sapienza University of Rome, 00185 Rome, Italy; giancarlo.ceccarelli@uniroma1.it; 4 Department of Biomedical Sciences, University of Sassari, 07100 Sassari, Italy; fscarpa@uniss.it

**Keywords:** Ebola virus disease, Ebola virus, hepatic injury, liver dysfunction, endothelial dysfunction, viral hemorrhagic fever, pathogenesis, immune dysregulation, cytokine storm, coagulopathy, aminotransferases, AST, ALT, prognosis, biomarkers, post-Ebola syndrome, supportive care, monoclonal antibodies, hepatocytes, Kupffer cells

## Abstract

**Background**: Ebola virus disease (EVD), caused by Ebola virus (*Orthoebolavirus zairense*), is one of the most lethal viral infections, with case fatality rates historically exceeding 80% in several outbreaks. Once classified as a viral hemorrhagic fever, EVD is now regarded as a systemic disorder with immune dysregulation, endothelial injury, metabolic impairment, and multiorgan dysfunction, in which hepatic involvement has been underappreciated. **Objective**: To synthesize the evidence on hepatic involvement in EVD (mechanisms of injury, clinical spectrum, prognostic significance, therapeutic implications, and long-term consequences in survivors) and to appraise its strength. **Methods**: Narrative review based on a structured, non-systematic PubMed search, with the search strategy reported in full and SANRA used as a reporting guide. **Results**: Hepatocytes and Kupffer cells are early targets of infection in human autopsy and animal studies. Hepatic injury reflects direct viral effects, macrophage activation, cytokine release, endothelial activation and barrier dysfunction, coagulopathy, and hypoperfusion, with mechanistic evidence largely derived from experimental models. Aminotransferase elevation, particularly of AST, is associated with severity and mortality but is not specific for liver injury (muscle injury, hemolysis, ischemia, and drugs also contribute). Monoclonal antibodies improve survival in disease caused by Ebola virus, whereas no approved specific therapy exists for Sudan or Bundibugyo virus disease. Subclinical hepatic and platelet abnormalities have been reported in survivors, but evidence for chronic liver disease is lacking. **Conclusions**: Hepatic dysfunction is an important, prognostically relevant component of EVD pathophysiology, interlinked with inflammatory and endothelial pathways. Whether it acts as a direct driver of disease progression, rather than a marker of systemic severity, remains to be established; prospective studies with systematic hepatological assessment are needed.

## Simple Summary

Ebola virus disease is a severe and often deadly illness caused by Ebola viruses. While much attention has focused on bleeding and shock, the liver also plays an important but underrecognized role. This review summarizes current knowledge on how Ebola virus affects the liver and blood vessels, and why this matters for patients. We found that the virus directly infects liver cells and triggers inflammation, blood vessel damage, and clotting problems. Liver injury is common and is linked to more severe disease and death, but it is not clear whether liver damage directly causes death or simply reflects how sick the patient is. Supportive care remains essential, and monoclonal antibody treatments are effective for one type of Ebola virus but not for others. Some survivors may have lasting liver abnormalities, though more research is needed. Understanding liver involvement in Ebola virus disease can help doctors better predict outcomes, tailor treatments, and improve long-term care for survivors. This knowledge is also valuable for responding to future outbreaks and other infectious diseases that affect multiple organs.

## 1. Introduction

Ebola virus disease (EVD) remains one of the most lethal viral infections known to humanity, with case fatality rates historically exceeding 80% in several major outbreaks [1,2]. Since the first recognized outbreaks in 1976 in the Democratic Republic of the Congo and Sudan, the virus has caused recurrent epidemics, predominantly in Central and West Africa, with the 2014–2016 West African outbreak representing the largest and most devastating in history, accounting for over 28,000 cases and 11,000 deaths [3,4]. The subsequent outbreaks in the Democratic Republic of the Congo (2018–2020) and the recent emergence of Sudan ebolavirus in Uganda (2022) and of Bundibugyo virus disease in the Democratic Republic of the Congo (2026) [5] have further underscored the persistent threat that Ebola viruses pose to global public health, particularly in regions with fragile healthcare infrastructure [6,7]. The continued occurrence of outbreaks, coupled with the logistical challenges of vaccine deployment in remote areas and the lack of approved specific therapeutics for Sudan virus disease and Bundibugyo virus disease (monoclonal antibodies are recommended by the World Health Organization (WHO) only for disease caused by Ebola virus), underscores the urgent need for a deeper understanding of the pathogenic mechanisms that drive disease severity and mortality [8].

Historically classified as a viral hemorrhagic fever, EVD is now understood as a systemic disorder characterized by profound immune dysregulation, endothelial injury, metabolic impairment, and progressive multiorgan dysfunction [9,10]. Within this evolving pathogenic framework, hepatic involvement appears substantially more relevant than previously appreciated. In traditional descriptions, liver injury was considered a secondary laboratory abnormality reflected by elevated aminotransferase levels, often overshadowed by the more dramatic manifestations of hemorrhage and shock [11]. However, accumulating evidence from clinical, pathological, and experimental studies indicates that hepatic dysfunction may represent an integral component of systemic collapse rather than a simple epiphenomenon [1,12,13]. This re-evaluation of the liver’s role in EVD pathogenesis has significant implications for understanding disease progression, identifying prognostic biomarkers, and developing targeted therapeutic strategies.

The liver appears to play an important role in the pathophysiology of EVD through multiple interconnected mechanisms. Experimental and pathological studies consistently demonstrate early viral dissemination in hepatic tissue, with hepatocytes and Kupffer cells among the principal cellular targets [13,14,15,16]. Viral replication within hepatocytes triggers a cascade of cellular stress responses, including endoplasmic reticulum stress, mitochondrial dysfunction, and activation of apoptotic pathways, ultimately leading to hepatocellular injury and necrosis [17,18]. At the same time, Ebola-associated liver injury cannot be explained solely by direct viral cytopathic effects. Hepatic alterations develop within a more complex context involving macrophage activation, inflammatory amplification, endothelial dysfunction, coagulation abnormalities, mitochondrial stress, metabolic derangement, and tissue hypoperfusion. Activation of Toll-like receptor 4 by the Ebola virus shed glycoprotein has been proposed as an initiating event in the inflammatory cascade (the evidence derives mainly from in vitro and animal studies) [19,20], directly linking viral components to the host innate immune response [21]. This inflammatory amplification is further driven by macrophage infection and activation, which contribute to the histopathological changes observed in target organs [22]. Endothelial activation and barrier dysfunction do not necessarily require productive endothelial infection, which appears to occur later in the disease course [13,23]. In critical cases, the clinical picture is complicated by vasodilatory shock, with specific immunologic correlates identified in nonhuman primate models [24]. Hepatic tissue itself exhibits a pronounced proinflammatory myeloid phenotype, characterized by the accumulation of activated myeloid cells that perpetuate local and systemic inflammation [25]. In vitro data suggest that viral modulation of transforming growth factor β signaling may induce markers of a mesenchyme-like transition in hepatocytes [17]; a functional or fibrotic consequence in patients has not been demonstrated. Ultimately, these interconnected pathways, i.e., inflammation, endothelial dysfunction, coagulopathy, and metabolic derangement, converge to produce the severe multiorgan failure characteristic of Ebola virus disease [26,27,28]. The liver’s dual role as both a target of infection and a critical regulator of systemic inflammation and coagulation creates a vicious cycle: viral replication in hepatocytes and Kupffer cells triggers the release of pro-inflammatory cytokines and chemokines, which in turn activate the hepatic acute phase response, promote endothelial activation, and perpetuate a systemic hyperinflammatory state that further compromises hepatic function [12,29,30]. This pathogenic cascade is amplified by the endothelial dysfunction characteristic of EVD, with the Ebola virus glycoprotein directly activating endothelial cells and disrupting vascular integrity, leading to increased vascular permeability, tissue edema, and impaired perfusion of vital organs including the liver [23,27,31,32].

The clinical spectrum of hepatic involvement in EVD ranges from mild transaminitis to acute liver failure, with elevated aminotransferase levels serving as consistent predictors of disease severity and mortality [33,34]. Patients with severe EVD frequently exhibit marked elevations in aspartate aminotransferase (AST) and alanine aminotransferase (ALT), often accompanied by prolonged prothrombin time and hypoalbuminemia, reflecting both hepatocellular damage and synthetic dysfunction [35,36]. These laboratory abnormalities correlate with the extent of hepatic viral replication and the intensity of the host inflammatory response, and they provide valuable prognostic information that can guide clinical management [37,38]. Understanding the clinical spectrum of hepatic involvement is therefore essential for risk stratification and the timely identification of patients who may benefit from intensive supportive care.

The prognostic significance of liver dysfunction in EVD is increasingly recognized, with mounting evidence that the degree of hepatic injury correlates with disease severity, the likelihood of progression to critical illness, and overall mortality [39,40]. Elevated AST and ALT levels at presentation have been consistently associated with fatal outcomes in both observational studies and outbreak cohorts, and these markers are now considered among the most reliable early predictors of disease severity [41,42]. Furthermore, the persistence of liver function abnormalities in survivors suggests that hepatic injury may have long-term consequences that extend beyond the acute phase of infection. Recent studies have reported subclinical hepatic laboratory abnormalities, including persistent liver enzyme or bilirubin abnormalities in some cohorts, months to years after acute infection, mostly in survivors of Sudan virus disease, raising questions about the durability of hepatic recovery and the potential for chronic liver disease in survivors [43,44,45]. These findings highlight the need for long-term monitoring of liver function in EVD survivors and underscore the importance of understanding the mechanisms that drive both acute and chronic hepatic injury.

The therapeutic and supportive implications of hepatic involvement in EVD are profound. The liver is central to drug metabolism, and hepatic dysfunction can significantly alter the pharmacokinetics and pharmacodynamics of experimental therapeutics, complicating dosing decisions and increasing the risk of drug toxicity [46,47]. Moreover, the coagulopathy and synthetic failure associated with severe hepatic injury necessitate careful management of fluid balance, coagulation parameters, and nutritional support [48]. The development of targeted therapies that address the underlying mechanisms of hepatic and endothelial injury, rather than merely managing their consequences, represents a promising avenue for improving outcomes in EVD. Several experimental approaches, including modulators of the host inflammatory response, inhibitors of viral entry and replication, and agents that stabilize endothelial barrier function, are currently under investigation and may offer new therapeutic options for patients with severe EVD [49,50,51].

Recent advances in transcriptomics, single-cell profiling, and experimental models have further expanded understanding of Ebola-associated organ injury [25,27,28,52]. High-resolution profiling of infected tissues has revealed the heterogeneity of cellular responses, the temporal dynamics of viral replication and host defense, and the critical role of myeloid cell populations in shaping the inflammatory milieu [27,28]. These technological advances have provided unprecedented insights into the molecular and cellular mechanisms that underlie hepatic and endothelial injury, and they offer new opportunities for the identification of therapeutic targets and the development of biomarkers for disease monitoring [53,54]. Survivor studies increasingly suggest that biological consequences may persist well beyond the acute viremic phase, with a substantial proportion of survivors experiencing post-Ebola syndrome characterized by chronic fatigue, arthralgia, ocular and auditory sequelae, and persistent inflammatory and metabolic abnormalities [43,44,45,55,56]. Notably, long-term subclinical hepatic laboratory abnormalities have been reported in some survivors, mainly of Sudan virus disease, months to years after initial infection, raising questions about the durability of hepatic recovery and the potential for chronic liver disease in EVD survivors.

Following current WHO nomenclature, “Ebola virus disease” (EVD) refers to disease caused by Ebola virus (EBOV; *Orthoebolavirus zairense*), whereas Sudan virus disease (SVD) and Bundibugyo virus disease (BVD) are caused by other ebolaviruses [5]. Most of the mechanistic, clinical, and therapeutic evidence discussed in this review derives from EBOV; data on SVD or BVD are identified explicitly, because pathogenesis, prognosis, and therapeutic options may differ and findings cannot be assumed to be interchangeable.

Liver injury during Ebola virus infection has recently been reviewed by Sui et al. [57], who summarized its characteristics and molecular mechanisms, including hepatic tropism, AST elevation, mild hyperbilirubinemia, impaired coagulation factor synthesis, hepatocyte necrosis, and inflammatory responses, and their relationship with disease severity and mortality. Hepatic involvement in emerging and exotic viral infections has also been addressed more broadly [1,12]. The present review complements these contributions in four respects. First, it analyzes hepatic injury in relation to endothelial activation and barrier dysfunction, explicitly separating infection-dependent from infection-independent mechanisms, and grades the strength of the evidence supporting each mechanism. Second, it critically appraises the specificity of aminotransferase abnormalities, including the extrahepatic and iatrogenic contributors to AST elevation. Third, it dedicates a section to survivors and post-Ebola syndrome, evaluating the evidence for persistent hepatic and hematological abnormalities and identifying the species (EBOV or SUDV) to which each finding refers. Fourth, it updates the therapeutic section according to current WHO recommendations, distinguishing EVD from SVD and BVD.

This narrative review is based on a structured, but not systematic, literature search and was prepared following the SANRA recommendations for narrative reviews [58]. Because the aim was to synthesize and critically appraise heterogeneous evidence (clinical, pathological, experimental, and mechanistic) rather than to answer a single focused question through quantitative synthesis, PRISMA methodology was not adopted. PubMed/MEDLINE was searched from inception to 31 August 2026. The search strategy was designed to identify studies relevant to hepatic involvement, endothelial dysfunction, immune dysregulation, disease severity, and long-term outcomes in EVD. The initial core search combined Ebola virus disease with terms related to hepatic involvement using the following search string: (“Ebola virus disease”[Title/Abstract] OR “Ebola”[Title/Abstract]) AND (liver OR hepatic OR hepatocyte OR hepatitis OR transaminases). Additional targeted searches were subsequently performed according to the main domains addressed in the review. Hepatic tropism and direct liver injury were investigated using (“Ebola” AND hepatocyte), (“Ebola virus” AND liver pathology), and (“Ebola” AND hepatic necrosis). Immune-mediated mechanisms and inflammatory dysregulation were explored using (“Ebola” AND cytokine storm), (“Ebola” AND immune dysregulation), and (“Ebola” AND inflammatory response). Endothelial dysfunction and vascular injury were assessed using (“Ebola” AND endothelial dysfunction), (“Ebola virus disease” AND vascular leakage), (“Ebola” AND endothelial injury), and (“Ebola” AND capillary leak). Searches addressing coagulation abnormalities included (“Ebola” AND coagulopathy), (“Ebola” AND disseminated intravascular coagulation), and (“Ebola” AND hemorrhagic manifestations). Prognostic markers and disease severity were investigated using (“Ebola” AND AST mortality), (“Ebola” AND transaminases prognosis), (“Ebola” AND biomarkers mortality), (“Ebola” AND viral load prognosis), and (“Ebola” AND severe disease predictors). Long-term outcomes and post-Ebola manifestations were explored using (“Post-Ebola syndrome” AND liver), (“Ebola survivors” AND inflammation), and (“Ebola survivors” AND long-term complications). Targeted searches addressing therapeutic and preventive interventions included (“Ebola” AND monoclonal antibodies), (“Ebola” AND Inmazeb), (“Ebola” AND Ebanga), (“Ebola” AND vaccine effectiveness), and (“rVSV-ZEBOV” AND Ebola). Relevant review articles were additionally identified using (“Ebola” AND liver) AND review[Publication Type] and (“Ebola virus disease” AND pathogenesis) AND review. The searches were limited to English-language publications. The combined searches yielded 150 records for initial assessment. Original experimental, pathological, translational, and clinical studies, together with relevant reviews and case reports, were considered according to their relevance to the aims of this narrative review. Reference lists of selected articles were also manually screened to identify additional pertinent publications. The final selection prioritized studies providing evidence on hepatic tropism and injury, macrophage and immune activation, endothelial dysfunction, coagulopathy, disease severity and prognosis, and post-Ebola sequelae. WHO guidance documents were consulted as grey literature for current recommendations [5,58]. Given the heterogeneity of the evidence, no formal risk-of-bias assessment was performed; instead, the strength of evidence supporting each mechanism or clinical statement was classified as established, probable, or hypothetical.

This review summarizes current evidence regarding hepatic involvement in EVD, structuring the available knowledge across several key domains. First, we examine the mechanisms of viral dissemination and the early events that establish hepatic infection, including the molecular interactions that facilitate viral entry into hepatocytes and Kupffer cells (Section 2). Second, we explore the interconnected pathways of endothelial and hepatic injury, emphasizing the role of inflammatory amplification, coagulopathy, and metabolic derangement in driving organ dysfunction (Section 3). Third, we characterize the clinical spectrum of hepatic involvement, from mild laboratory abnormalities to acute liver failure (Section 4). Fourth, we discuss the prognostic significance of liver dysfunction and its utility as a marker of disease severity (Section 5). Fifth, we consider the therapeutic and supportive implications of hepatic injury, including the challenges of drug dosing and the management of coagulopathy (Section 6). Sixth, we examine the long-term consequences of hepatic involvement, including the emerging evidence of persistent liver dysfunction in EVD survivors (Section 7). Finally, we critically discuss to what extent the available evidence supports viewing EVD as a multisystem disease in which endothelial and hepatic dysfunction interact through inflammatory, vascular, metabolic, and immunological pathways, distinguishing association from causation, and we summarize the key findings and their implications for clinical practice and future research (Section 8 and Section 9). A comprehensive understanding of these mechanisms is essential for improving clinical management, developing targeted therapeutic strategies, and addressing the long-term health consequences in EVD survivors. Throughout the manuscript, we distinguish evidence relating to Ebola virus disease from that relating to Sudan and Bundibugyo virus diseases, and we classify the strength of evidence for key mechanisms as established, probable, or hypothetical.

## 2. Viral Dissemination and Endothelial–Hepatic Injury

**Figure 1:**
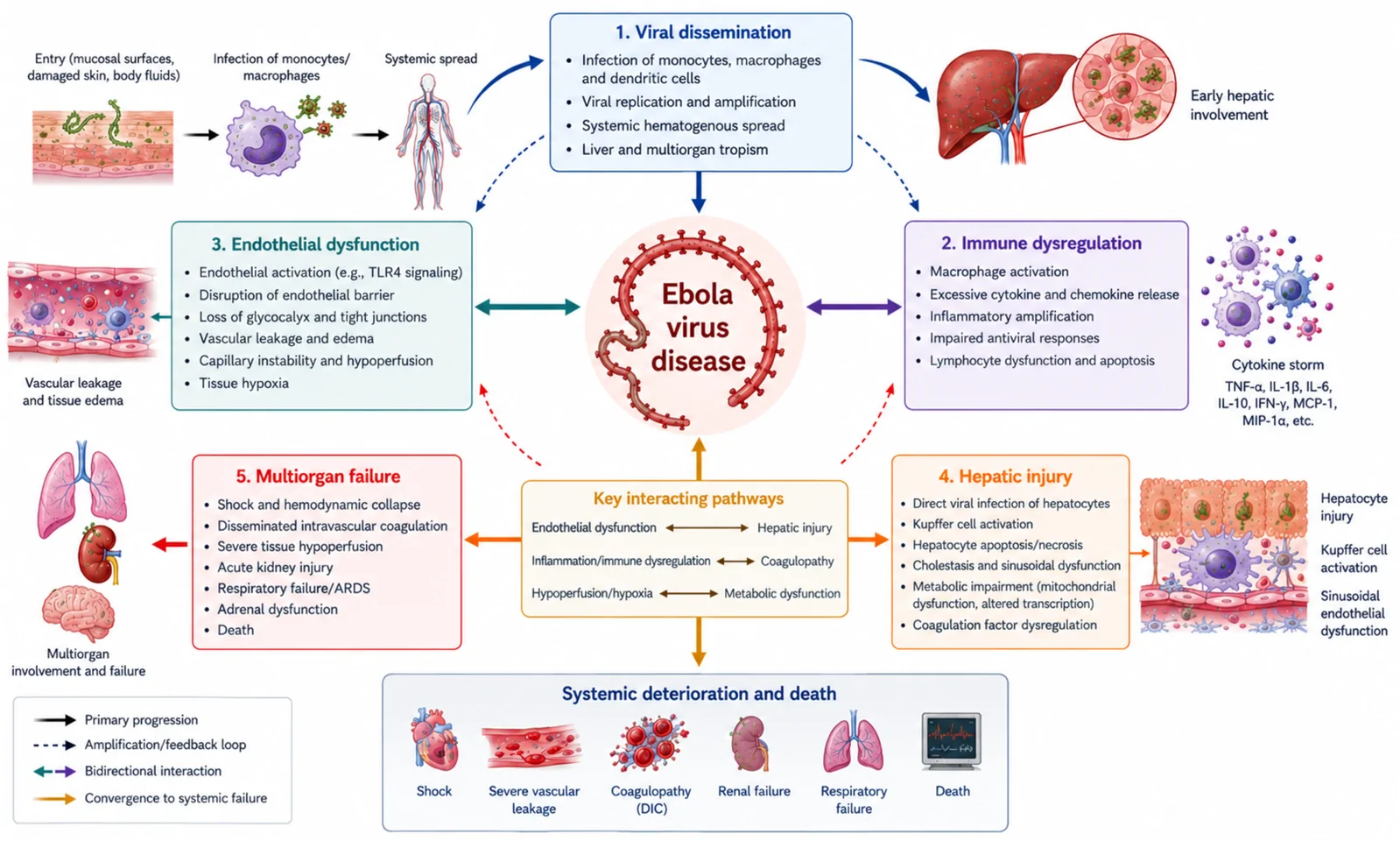
**Pathogenic model of endothelial–hepatic dysfunction in Ebola virus disease**. Ebola virus disseminates to the liver early during infection, infecting hepatocytes and Kupffer cells. Direct viral cytopathic effects, combined with macrophage activation and inflammatory amplification, trigger endothelial dysfunction, vascular leakage, and coagulation abnormalities. Hepatic dysfunction contributes to systemic deterioration through impaired coagulation factor synthesis, metabolic dysregulation, and reduced inflammatory control. These interconnected mechanisms ultimately lead to multiorgan failure. Key mediators include glycoprotein (GP)-induced endothelial activation via Toll-like receptor 4 (TLR4), cytokine release (IL-6, TNF-α, MCP-1, MIP-1α), and disruption of vascular integrity. Abbreviations: ARDS, acute respiratory distress syndrome; DIC, disseminated intravascular coagulation; GP, glycoprotein; IFN, interferon; IL, interleukin; MCP-1, monocyte chemoattractant protein-1; MIP-1α, macrophage inflammatory protein-1 alpha; TLR4, Toll-like receptor 4; TNF-α, tumor necrosis factor alpha; EVD, Ebola virus disease. Endothelial dysfunction (box 3) refers mainly to infection-independent endothelial activation and barrier disruption mediated by glycoprotein, TLR4 signaling, and cytokines; productive endothelial infection is considered a later and less well characterized event. The model is conceptual, and the strength of evidence differs between its components.

As illustrated in Figure 1, EVD can be conceptualized as a multisystem disorder in which viral dissemination, immune dysregulation, endothelial dysfunction, coagulation abnormalities, and hepatic injury are tightly interconnected and mutually reinforcing, ultimately leading to systemic deterioration, multiorgan failure, and death [1,2,9].

The pathogenic cascade is initiated by the entry of Ebola virus through mucosal surfaces, skin breaches, or parenteral exposure, followed by rapid infection of monocytes, macrophages, and dendritic cells at the site of entry and within regional lymph nodes [9,10]. These early infected cells serve as both viral amplification hubs and primary drivers of the initial inflammatory response, producing large quantities of pro-inflammatory cytokines and chemokines that recruit additional immune cells and propagate the inflammatory cascade [22,29]. From these primary replication sites, the virus disseminates hematogenously to secondary target organs, with the liver representing one of the earliest and most severely affected organs [13,14,15,16]. The liver is a major target of Ebola virus infection, with hepatocytes and Kupffer cells serving as principal sites of viral replication [13,14,16]. Following systemic spread, Ebola virus gains entry into hepatocytes through multiple receptor-mediated mechanisms, including interaction with T-cell immunoglobulin and mucin domain 1 (TIM-1), Tyro3, Axl, and Mer (TAM) receptors, and the endosomal protein Niemann-Pick C1 (NPC1) [15,16]. Once inside the cell, the virus exploits the host endocytic machinery to deliver its nucleocapsid into the cytoplasm, where replication and transcription occur in specialized viral factories [8,10]. Productive infection of hepatocytes results in the release of large quantities of progeny virions into the sinusoidal space, perpetuating local spread and contributing to the high viral loads observed in severe disease [8]. Kupffer cells, the tissue-resident macrophages of the liver, are also efficiently infected and, upon activation, release a barrage of inflammatory mediators that amplify the local inflammatory response [22]. The dual infection of both hepatocytes and Kupffer cells establishes a self-perpetuating cycle of viral replication, cellular injury, and inflammatory amplification that drives progressive hepatic dysfunction [25,28].

A hallmark of severe EVD is widespread endothelial dysfunction, which contributes to the vascular leakage, tissue edema, and impaired organ perfusion characteristic of the disease [23,31,32]. The Ebola virus glycoprotein (GP) plays a central role in this process, activating endothelial cells through several mechanisms that do not necessarily require productive endothelial infection. The shed GP, which is released in large quantities from infected cells, activates Toll-like receptor 4 (TLR4) signaling, triggering a cascade of pro-inflammatory responses that compromise endothelial barrier function [21]. This finding derives mainly from in vitro and animal experiments [59], and its relevance to human disease remains to be established. Additionally, GP expressed on the surface of infected cells or incorporated into virions interacts with endothelial cell surface receptors, activating host cytoskeletal signaling pathways that lead to the disruption of adherens and tight junctions [60]. This disruption of intercellular junctions increases vascular permeability, allowing fluid and macromolecules to leak into the interstitial space, resulting in tissue edema and hypovolemia [31,32]. The resulting endothelial activation is characterized by upregulation of adhesion molecules such as ICAM-1 and VCAM-1, which promote the recruitment of leukocytes and further amplify the inflammatory response [23]. Endothelial activation and barrier dysfunction should be distinguished from endothelial infection. Cytokines released by infected or glycoprotein-stimulated macrophages and dendritic cells can increase endothelial permeability without infection of endothelial cells [23,31], whereas productive endothelial infection has mainly been described at late stages of disease in human tissues and non-human primates [13,23] and is therefore more likely to amplify than to initiate vascular injury. The relative contribution of infection-dependent and infection-independent mechanisms to vascular leakage in patients has not been quantified. Simultaneously, the immune response to Ebola virus infection is characterized by a paradoxical combination of excessive inflammation and impaired antiviral defense [9,26]. Macrophages and dendritic cells, upon infection, produce large quantities of pro-inflammatory cytokines, including IL-6, TNF-α, MCP-1, and MIP-1α, creating a “cytokine storm” that drives the systemic inflammatory response syndrome [29,30]. This hyperinflammatory state is accompanied by impaired type I interferon responses, which are critical for limiting viral replication and establishing an effective antiviral state [18,28]. The combination of excessive inflammation and deficient interferon signaling creates a permissive environment for unchecked viral replication and dissemination [25]. Concurrently, profound lymphocyte dysfunction and apoptosis contribute to the immunosuppression that characterizes severe EVD, with lymphocyte depletion observed in the spleen, lymph nodes, and peripheral blood [30]. The coagulopathy of EVD is a direct consequence of both endothelial dysfunction and the inflammatory cascade [26]. Endothelial activation leads to the exposure of tissue factor, which triggers the extrinsic coagulation pathway, resulting in consumptive coagulopathy and disseminated intravascular coagulation [26,54]. Simultaneously, hepatic dysfunction contributes to coagulopathy through impaired synthesis of coagulation factors, including factors II, V, VII, IX, and X, and reduced production of natural anticoagulants such as protein C and antithrombin III [1,12].

Hepatic dysfunction in EVD arises from the convergence of direct viral injury, inflammatory damage, and ischemic insult [12,13]. Direct viral infection of hepatocytes induces cell stress responses, including endoplasmic reticulum stress, mitochondrial dysfunction, and activation of apoptotic pathways, resulting in hepatocyte necrosis and the release of damage-associated molecular patterns that further amplify inflammation [17,18]. These stress pathways have been characterized mainly in cell-culture and animal models, and direct evidence in human liver tissue is limited. Kupffer cell activation contributes to the inflammatory milieu through the production of TNF-α, IL-1β, and reactive oxygen species, which can induce hepatocyte injury through both direct and indirect mechanisms [22]. The resulting impairment of sinusoidal perfusion, due to endothelial dysfunction and microthrombi formation, exacerbates hypoxic injury to hepatocytes [25,28]. The systemic impact of hepatic dysfunction is profound: the liver is responsible for the synthesis of most coagulation factors, and its impairment contributes directly to the coagulopathy of EVD [1]. The reduced synthesis of albumin and other plasma proteins compromises oncotic pressure and contributes to the development of edema [12]. Hepatic metabolic dysfunction, including impaired gluconeogenesis and altered lipid metabolism, contributes to the metabolic derangements observed in severe EVD [18]. Furthermore, the liver’s role in the clearance of inflammatory mediators and endotoxins is compromised, allowing these factors to accumulate in the circulation and perpetuate systemic inflammation [12,29]. The convergence of these interconnected pathways, i.e., viral dissemination, endothelial dysfunction, immune dysregulation, coagulopathy, and hepatic injury, produces the progressive multiorgan failure that characterizes fatal EVD [9,10,27]. The systemic hyperinflammatory state drives vasodilatory shock, characterized by hypotension, decreased systemic vascular resistance, and impaired tissue perfusion [24]. The combination of shock, coagulopathy, and vascular leakage leads to tissue hypoxia, acute kidney injury, and respiratory failure [26,48]. Adrenal dysfunction, resulting from direct viral infection and inflammatory injury to the adrenal gland, further compromises hemodynamic stability and impairs the stress response [28]. The convergence of these pathogenic mechanisms creates a vicious cycle of deterioration, in which each component exacerbates the others, ultimately leading to irreversible multiorgan failure and death [2,9,27].

## 3. Mechanisms of Hepatic and Systemic Injury

Direct infection of hepatocytes represents a principal mechanism underlying liver injury during EVD, with the virus establishing productive replication in these cells shortly after systemic dissemination [13,14,16]. The multiple mechanisms contributing to hepatic injury, including direct viral cytopathic effects, macrophage activation, endothelial dysfunction, coagulopathy, immune dysregulation, and metabolic disturbances, are detailed in Table 1.

Histopathological studies consistently demonstrate extensive viral dissemination in hepatic tissue with hepatocellular degeneration, necrosis, sinusoidal disruption, and inflammatory infiltration [6,12,13,25,26]. In experimental models, viral replication within hepatocytes triggers a cascade of cellular stress responses, including endoplasmic reticulum stress, mitochondrial dysfunction, and activation of apoptotic pathways, ultimately leading to hepatocellular injury and necrosis [17,18]. In vitro data suggest that viral modulation of transforming growth factor β signaling may induce markers of a mesenchyme-like transition in hepatocytes [17]; this remains a hypothesis, as a functional or fibrotic consequence has not been demonstrated in patients.

Severe disease is characterized by profound inflammatory dysregulation. Ebola virus infection induces excessive activation of innate immune pathways associated with uncontrolled cytokine release, impaired antiviral coordination, and diffuse inflammatory signaling [15,16]. Activated monocytes, macrophages, and dendritic cells release inflammatory mediators, including IL-6, TNF-α, MCP-1, and MIP-1α, that are capable of amplifying tissue injury across multiple organ systems [29,30]. In experimental systems, shed glycoproteins activate Toll-like receptor 4 signaling [19,20,21], while VP40 has been implicated in inflammatory responses associated with severe disease and virulence [2,24]. The inflammatory response appears maladaptive rather than simply exaggerated, characterized by coexistence of ineffective antiviral control and excessive inflammatory activation [9,26]. Within the liver, this environment promotes metabolic dysfunction, endothelial instability, mitochondrial stress, and progressive tissue injury [18,25].

Endothelial dysfunction is a defining feature of severe EVD and explains many catastrophic clinical manifestations [23,27,31]. Ebola glycoprotein directly disrupts endothelial integrity through host cytoskeletal signaling, resulting in increased vascular permeability [14,60]. Importantly, these mechanisms (glycoprotein-mediated activation and cytokine-induced barrier disruption) do not require productive infection of endothelial cells, which occurs later in the disease course and is less well characterized [13,23,31]; Table 1 therefore lists endothelial activation/barrier dysfunction and endothelial cell infection as separate entries. Extracellular vesicles released during infection may propagate inflammatory and endothelial signaling beyond sites of viral replication [61]. Clinically, these alterations manifest as capillary leakage, hypotension, tissue edema, impaired organ perfusion, and progressive circulatory instability [8,62]. In advanced disease, severe endothelial injury contributes to vasodilatory shock and widespread tissue hypoperfusion [24]. Coagulation abnormalities frequently coexist with endothelial dysfunction, reflecting combined effects of vascular injury, inflammatory activation, macrophage signaling, and hepatic impairment [26,54]. Severe disease may evolve toward disseminated intravascular coagulation-like states characterized by thrombocytopenia, prolonged coagulation parameters, diffuse microvascular injury, and refractory shock [8,62]. Reduced hepatic perfusion, sinusoidal injury, impaired coagulation factor synthesis, and metabolic collapse may further aggravate endothelial instability and multiorgan failure [1,12].

**Table 1:** Principal mechanisms contributing to hepatic injury during Ebola virus disease. Evidence level: E, established; P, probable; H, hypothetical. Abbreviations: EVD, Ebola virus disease; TIM-1, T-cell immunoglobulin and mucin domain 1; NPC1, Niemann–Pick C1; ER, endoplasmic reticulum; UPR, unfolded protein response; TNF-α, tumor necrosis factor alpha; IL, interleukin; IFN, interferon; NF-κB, nuclear factor kappa B; VEGF, vascular endothelial growth factor; NO, nitric oxide; DIC, disseminated intravascular coagulation; ROS, reactive oxygen species; VP, viral protein; GP, glycoprotein; TLR4, Toll-like receptor 4; NHP, non-human primate.

Mechanism	Biological Basis	Hepatic Consequence	Key Molecular/Cellular Pathways	Evidence Level	References
Direct viral cytopathic injury	Infection and replication of Ebola virus in hepatocytes	Focal and diffuse hepatocellular necrosis	The virus binds to surface receptors (e.g., TIM-1, NPC1), activates viral polymerase, and triggers apoptosis via both extrinsic (caspase-8) and intrinsic (mitochondrial) pathways. Massive replication overwhelms the endoplasmic reticulum, inducing ER stress and the UPR response, ultimately culminating in cell death. Viral modulation of TGF-β signaling may promote a mesenchyme-like transition in hepatocytes.	Hepatocyte infection and necrosis: E (human autopsy, NHP). ER stress and apoptosis pathways: P (in vitro, animal). Mesenchyme-like transition: H (in vitro only).	[6,13,17,25,26]
Macrophage activation and cytokine storm	Massive release of pro-inflammatory cytokines from infected macrophages and Kupffer cells	Inflammatory hepatitis with mixed cellular infiltrate	Infected macrophages secrete TNF-α, IL-1β, IL-6, IL-18, and IFN-γ, which amplify the recruitment of neutrophils and lymphocytes. This creates a toxic microenvironment for hepatocytes, with oxidative damage and amplification of NF-κB signaling. The cytokine storm correlates with disease severity.	Association of cytokine levels with severity: E (human cohorts). Hepatocellular toxicity via NF-κB: P.	[9,12,15,16,27,29,30]
Endothelial activation and barrier dysfunction (infection-independent)	Glycoprotein- and cytokine-mediated activation of endothelial cells, not necessarily requiring productive infection	Hepatic hypoperfusion and ischemic/metabolic injury	Cytokines (VEGF, TNF-α) reduce the expression of tight junctions (occludin, claudin-5) and increase the production of NO and prostacyclin, causing vasodilation and fluid loss. GP activates endothelial cells and disrupts endothelial integrity through host cytoskeletal signaling; shed GP may act via TLR4. This results in reduced portal and arterial flow, leading to tissue hypoxia and lactate accumulation.	Capillary leak: E (clinical). Molecular mediators (GP, TLR4, VEGF, junction proteins): P (in vitro, animal).	[14,19,20,21,23,31,32,60,62]
Endothelial cell infection	Productive infection of endothelial cells, mainly at late stages	Amplification of vascular injury and hemorrhage in advanced disease	Considered a late event that may amplify, rather than initiate, barrier dysfunction; its contribution to vascular leakage in patients has not been quantified.	P (human autopsy, NHP).	[13,23]
Coagulopathy (DIC-like)	Activation of coagulation and consumption of clotting factors	Sinusoidal dysfunction and microthrombosis	Tissue factor activation by infected macrophages and endothelial cells triggers the coagulation cascade, with consumption of fibrinogen and platelets. Microthrombi in the hepatic sinusoids are thought to obstruct blood flow, exacerbating hypoxia and promoting patchy necrosis. Fibrinogenesis contributes to bleeding, but at the hepatic level, microvascular thrombosis is considered to predominate.	Coagulopathy/DIC: E (clinical). Tissue factor-driven mechanism: P (NHP). Hepatic sinusoidal microthrombosis: H (limited human histology).	[8,26,54,62]
Immune dysregulation	Ineffective antiviral response and persistent inflammation	Chronic inflammatory damage and early fibrosis	Infection impairs dendritic cells and T lymphocytes, reducing type I IFN production. Simultaneously, M1-polarized macrophages maintain a sustained inflammatory state with persistent release of IL-6 and TNF-α. This imbalance prevents viral clearance and favors ongoing tissue damage, with possible evolution toward periportal fibrosis in survivors (speculative).	Lymphocyte apoptosis and impaired interferon response: E/P. Periportal fibrosis in survivors: H (no supporting data).	[13,15,16,18,28,30]
Metabolic/mitochondrial dysfunction	Alterations in oxidative phosphorylation and gene transcription	Disrupted hepatic homeostasis (steatosis, altered glucose metabolism)	Viral proteins (e.g., VP24, VP35) may interfere with respiratory chain complexes, reducing ATP production and increasing ROS. In parallel, the expression of genes involved in gluconeogenesis and β-oxidation is suppressed, leading to triglyceride accumulation in hepatocytes (microvesicular steatosis) and relative hyperglycemia. This metabolic disturbance may contribute to the severity of the clinical picture.	Steatosis at autopsy: E (descriptive). Downregulation of hepatic genes: P (iPSC-derived hepatocytes, NHP). Viral-protein-mediated respiratory chain inhibition and its causal role: H.	[10,17,18,25,28]

Profound immune dysregulation is another hallmark of advanced EVD. Severe infection is characterized by dysfunctional innate immunity, impaired antiviral responses, extensive lymphocyte apoptosis, and persistent inflammatory signaling [13,15,16]. Bradfute et al. demonstrated marked lymphocyte apoptosis during Ebola infection, likely contributing to ineffective adaptive immune responses and impaired viral control [13]. Transient liver injury has also been associated with impaired glycoprotein-specific antibody responses and lymphopenia, suggesting a relationship between hepatic dysfunction and defective antiviral immunity [14]. This immune dysregulation is further characterized by impaired type I interferon responses, which are critical for limiting viral replication and establishing an effective antiviral state [18,28]. Concurrently, M1-polarized macrophages maintain a sustained inflammatory state with persistent release of IL-6 and TNF-α, preventing viral clearance and favoring ongoing tissue damage [25,30].

Hepatic dysfunction reflects combined effects of viral cytopathic damage, inflammatory dysregulation, endothelial instability, metabolic failure, tissue hypoperfusion, and immune collapse occurring simultaneously during severe systemic disease [9,12,27]. The principal mechanisms contributing to hepatic injury, along with their biological basis, hepatic consequences, and key molecular pathways, are detailed in Table 1. This integrated view underscores the complex interplay between viral replication, host immune responses, and organ-specific injury that characterizes severe EVD [2,9,27]. Understanding these interconnected mechanisms is essential for developing targeted therapeutic strategies and identifying biomarkers for disease progression and prognosis [49,50,51].

## 4. Clinical Spectrum of Hepatic Involvement

Abnormal liver biochemistry is frequently observed during Ebola virus disease and likely reflects the combined effects of direct hepatic injury, systemic inflammation, endothelial dysfunction, and tissue hypoperfusion [1,4,12]. The main biochemical and clinical manifestations associated with hepatic involvement, along with their pathophysiological implications and mechanistic pathways, are summarized in Table 2.

Elevated aminotransferases, particularly aspartate aminotransferase (AST), represent some of the most consistent laboratory abnormalities reported in severe disease [1,6,16]. Interestingly, the predominance of AST over alanine aminotransferase (ALT) differs from the biochemical profile typically associated with classical acute viral hepatitis. This pattern probably reflects the multisystem nature of EVD rather than isolated hepatocellular necrosis alone. In severe disease, AST elevation may derive not only from hepatic injury, but also from skeletal muscle damage, endothelial disruption, systemic inflammation, mitochondrial dysfunction, and ischemic tissue injury related to circulatory instability [1,9,16]. AST elevation is therefore not specific for hepatic injury, and its interpretation requires consideration of the extrahepatic and iatrogenic sources summarized in Table 2. Clinical studies have consistently demonstrated that elevated AST levels at presentation are associated with fatal outcomes, with AST elevation often preceding other markers of organ dysfunction [33,35,37].

Hyperbilirubinemia is generally less prominent and tends to occur later during advanced stages characterized by severe hepatocellular dysfunction or multiorgan failure [1,12]. When present, elevated total and direct bilirubin reflects dysfunction of sinusoidal and canalicular transporters due to cytokine-mediated cholestasis and direct viral injury to biliary epithelia [1]. Hypoalbuminemia may also develop as a consequence of impaired hepatic synthesis, systemic inflammation, capillary leakage, and critical illness physiology [9,16]. Coagulation abnormalities are common and may include prolonged prothrombin time, elevated international normalized ratio (INR), thrombocytopenia, and disseminated intravascular coagulation-like manifestations [9,16,48]. Importantly, these laboratory alterations may appear relatively early during infection, sometimes preceding overt hemorrhagic manifestations or advanced organ dysfunction [25,26]. In this context, liver test abnormalities should probably be interpreted less as isolated hepatic findings and more as part of a broader endothelial–inflammatory syndrome [9,27].

Aminotransferase elevation in EVD is not specific for hepatic injury and may have several sources. Skeletal muscle injury, including rhabdomyolysis, may raise AST, and to a lesser extent ALT, independently of hepatocellular damage; measurement of creatine kinase (with creatinine and potassium when myoglobinuria is suspected) helps to identify this contribution [1,9,16]. Cardiac and renal injury and systemic mitochondrial dysfunction may also contribute to AST release. Hemolysis, which has been reported alongside transient liver damage [14], can increase AST and lactate dehydrogenase and produce an unconjugated (indirect) hyperbilirubinemia, so that bilirubin fractionation and assessment of lactate dehydrogenase, haptoglobin, and blood counts are informative; in vitro hemolysis of samples collected under outbreak conditions may also spuriously increase AST. Ischemic (hypoxic) hepatitis secondary to shock produces an abrupt and marked rise in aminotransferases and lactate dehydrogenase that typically regresses rapidly once perfusion is restored [16,48]. Drug-induced liver injury is a further confounder: patients are frequently exposed to investigational or repurposed antivirals, antimalarials, antibiotics, and antipyretics [38,46], and a temporal relationship with drug exposure, together with formal causality assessment when feasible, should be sought before attributing enzyme changes to the disease itself. Finally, co-infections that are common in endemic settings (for example, malaria and viral hepatitis) and pre-existing liver disease may contribute. In practice, ALT, the AST/ALT ratio, bilirubin fractionation, INR, and albumin, interpreted together with creatine kinase and lactate dehydrogenase, provide a more reliable picture of hepatic involvement than AST alone.

The clinical spectrum of hepatic involvement in EVD is highly heterogeneous, ranging from mild transient transaminase elevation to severe hepatic dysfunction associated with shock physiology and multiorgan failure (Table 3) [1,12,16]. In some patients, liver injury may remain relatively subtle and clinically limited. In others, progressive hepatocellular dysfunction contributes to impaired metabolic homeostasis, coagulation disturbances, inflammatory amplification, and hemodynamic instability during advanced stages of disease [6,9,16]. Histopathological studies have demonstrated hepatocellular degeneration, necrosis, sinusoidal congestion, inflammatory infiltrates, and diffuse tissue injury involving multiple hepatic compartments [4,26]. These alterations may impair not only hepatic synthetic function, but also intrahepatic immune regulation and metabolic adaptation during severe systemic stress [25,28].

Importantly, liver dysfunction in EVD rarely behaves like a classic isolated viral hepatitis syndrome. Instead, it usually develops within a much broader clinical picture characterized by endothelial injury, vascular leakage, inflammatory dysregulation, coagulation abnormalities, and widespread organ involvement [9,27]. In critically ill patients, hepatic injury frequently coexists with circulatory collapse, renal dysfunction, respiratory failure, severe metabolic disturbances, and immune dysregulation [9,16,48]. Wolf et al. described a patient with extensive vascular leakage, refractory shock, metabolic derangement, and multiorgan failure requiring advanced intensive care support [16]. Similar findings have also emerged from experimental critical care models [9,24]. Within this context, the liver may actively contribute to systemic deterioration through impaired coagulation factor synthesis, altered inflammatory regulation, reduced detoxification capacity, metabolic dysfunction, and decreased albumin production [1,12]. As disease severity progresses, the distinction between “hepatic involvement” and “systemic disease” becomes increasingly difficult to separate [9,27].

The prognostic significance of liver dysfunction is increasingly recognized, with mounting evidence that the degree of hepatic injury correlates with disease severity and overall mortality [39,40,41]. Elevated AST and ALT levels at presentation have been consistently associated with fatal outcomes in both observational studies and outbreak cohorts, and these markers are now considered among the most reliable early predictors of disease severity [33,35,36,37]. The presence of thrombocytopenia, prolonged INR, and hypoalbuminemia further portends a poor prognosis, reflecting the convergence of hepatic synthetic failure and consumptive coagulopathy [16,48].

## 5. Prognostic Significance of Liver Dysfunction

**Figure 2:**
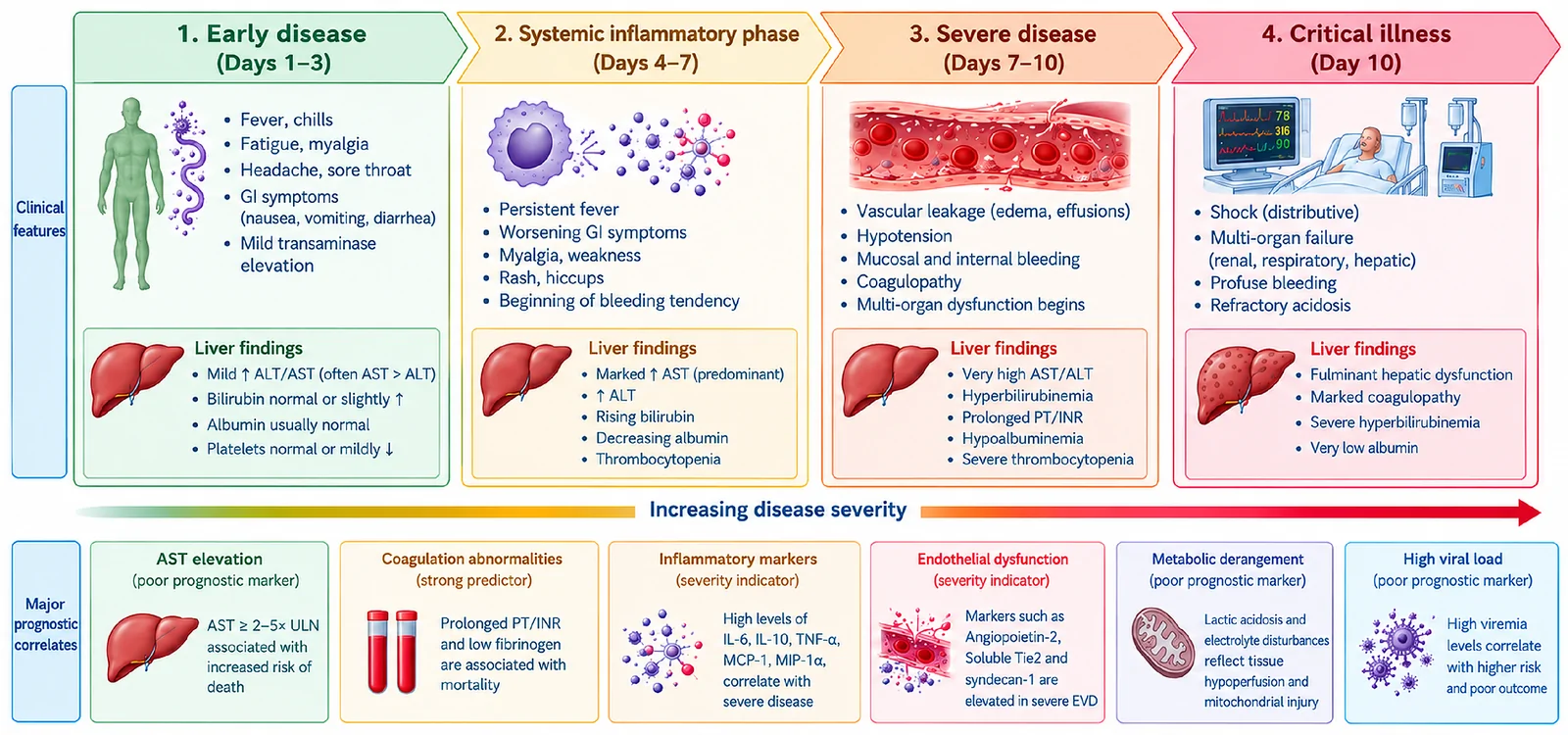
**Clinical progression and prognostic correlates of hepatic involvement in Ebola virus disease**. Liver dysfunction evolves within a broader systemic syndrome characterized by inflammatory activation, endothelial injury, coagulation abnormalities, metabolic dysfunction, and progressive multiorgan involvement. AST elevation, coagulation abnormalities, vascular instability, and inflammatory biomarkers are consistently associated with severe disease and adverse outcomes. Abbreviations: ALT, alanine aminotransferase; AST, aspartate aminotransferase; ARDS, acute respiratory distress syndrome; EVD, Ebola virus disease; GI, gastrointestinal; IL, interleukin; INR, international normalized ratio; MCP-1, monocyte chemoattractant protein-1; MIP-1α, macrophage inflammatory protein-1 alpha; PT, prothrombin time; TNF-α, tumor necrosis factor alpha; ULN, upper limit of normal. Time windows are approximate.

Accumulating evidence suggests hepatic dysfunction is closely associated with disease severity and adverse outcomes in Ebola virus disease. The clinical evolution and major prognostic correlates of hepatic involvement are illustrated in Figure 2, which highlights the key laboratory abnormalities and clinical features associated with disease progression and adverse outcomes.

Liver injury reflects the overall burden of viral dissemination, inflammatory activation, endothelial dysfunction, metabolic failure, and progressive multiorgan involvement [6,9,15,16]. The clinical progression of EVD is characterized by a recognizable sequence of phases, each associated with distinct laboratory findings and prognostic implications, as summarized in Figure 2. In brief, mild transaminase elevation with fever and gastrointestinal symptoms in the first days is followed by progressive liver enzyme elevation and a bleeding tendency (approximately days 4–7), by marked AST/ALT elevation, hyperbilirubinemia, prolonged PT/INR, vascular leakage, and coagulopathy in severe disease (approximately days 7–10), and by shock, multiorgan failure, and fulminant hepatic dysfunction in critical illness (beyond day 10). These time windows are approximate and vary between patients.

Experimental studies show severe disease is associated with higher levels of hepatic viral replication, broader transcriptional dysregulation, and more extensive inflammatory infiltration [15,25,26,28]. Clinically, patients with severe EVD frequently develop marked aminotransferase elevation, coagulation abnormalities, progressive hemodynamic instability, and metabolic dysfunction [9,16,29]. The severity of hepatic involvement correlates with the magnitude of viral replication, the intensity of the host inflammatory response, and the extent of endothelial dysfunction [25,27]. Among laboratory abnormalities, AST elevation has emerged as one of the markers most consistently associated with severe disease and mortality [1,6,16,33,37]. Marked elevation reflects combined effects of hepatocellular damage, systemic inflammation, endothelial dysfunction, skeletal muscle injury, mitochondrial stress, and tissue hypoperfusion [9,16,18]. Observational studies have consistently demonstrated that AST levels exceeding 2–5 times the upper limit of normal are associated with a significantly increased risk of death [33,35,37]. These are associations derived from observational data, and AST also reflects non-hepatic injury (Table 2); current evidence does not establish whether hepatic injury contributes causally to mortality or mainly marks the overall severity of systemic disease, and no AST threshold has been validated to guide treatment decisions. Coagulation abnormalities, particularly prolonged PT/INR and low fibrinogen, represent another strong predictor of mortality [9,16,26,48]. The prognostic value of these markers is further enhanced when considered in combination, as the convergence of hepatic synthetic failure, consumptive coagulopathy, and systemic inflammation identifies patients at the highest risk of adverse outcomes [16,48].

Inflammatory markers, including IL-6, IL-10, TNF-α, MCP-1, and MIP-1α, are consistently elevated in severe EVD and correlate with disease severity and mortality [27,29,30]. These cytokines reflect the intensity of the host inflammatory response and the degree of immune dysregulation, and they provide valuable prognostic information that complements traditional biochemical markers [9,26]. Endothelial dysfunction markers, such as Angiopoietin-2, soluble Tie2, and syndecan-1, are also elevated in severe EVD and reflect the extent of vascular injury and barrier disruption [24,31]. Metabolic derangement, including lactic acidosis and electrolyte disturbances, reflects tissue hypoperfusion and mitochondrial injury, and has been consistently associated with poor outcomes [9,48]. High viral load, as measured by PCR, is another strong predictor of mortality, with higher viremia levels correlating with more extensive hepatic injury and worse outcomes [8,16]. The combination of these prognostic markers, i.e., AST elevation, coagulation abnormalities, inflammatory markers, endothelial dysfunction markers, metabolic derangement, and high viral load, provides a comprehensive picture of disease severity and helps identify patients at highest risk of adverse outcomes [9,27].

Recent advances in biomarker analysis and computational modeling have improved understanding of prognostic determinants [59,63]. Combinations of inflammatory, hematologic, endothelial, metabolic, and hepatic markers may help identify patients at increased risk of adverse outcomes [9,15,29,63]. Machine learning-based prognostic models integrating dynamic biomarker assessment may eventually facilitate earlier escalation of supportive care and more individualized therapeutic strategies [63]. Nevertheless, disease severity in EVD is unlikely to depend on any single parameter in isolation. Clinical outcomes probably reflect the cumulative interaction between viral burden, immune responses, endothelial dysfunction, vascular instability, metabolic collapse, and progressive organ injury [9,16,27,28]. A multiparametric approach that integrates clinical, laboratory, and biomarker data is therefore essential for accurate risk stratification and for guiding clinical management decisions [9,48].

## 6. Therapeutic and Supportive Implications

Supportive care remains a principal determinant of survival in severe EVD, with comprehensive critical care interventions significantly reducing mortality when implemented early and systematically [8,62] Hepatic injury, endothelial dysfunction, vascular leakage, inflammatory amplification, and metabolic failure are closely interconnected, requiring systemic rather than organ-specific management [7,8,16,53]. The recognition that EVD is a multisystem disorder has shifted the therapeutic paradigm from isolated symptom management toward integrated organ support and hemodynamic optimization [27,47].

Patients with severe EVD develop hypovolemia, electrolyte disturbances, metabolic acidosis, capillary leakage, and circulatory instability driven by gastrointestinal fluid losses and endothelial disruption [8,47]. Maintaining tissue perfusion is essential for hemodynamic stabilization and limiting secondary organ injury, particularly in the liver, where hypoperfusion exacerbates existing hepatocellular damage [8,11,27]. The World Health Organization guidelines for the clinical management of filovirus disease emphasize systematic clinical and laboratory monitoring, oral and intravenous fluid therapy, management of shock, and use of vasopressors as cornerstones of patient-centred supportive care [58]. Oral rehydration is recommended in patients who can drink, while parenteral administration of appropriate fluids is indicated in those unable to drink or with severe dehydration, sepsis, or shock [58]. The WHO guidelines advise early, targeted use of intravenous fluids and vasoactive medications for treating shock, with norepinephrine as the preferred vasopressor agent when blood pressure support is needed [58]. Resuscitation with intravenous or intraosseous fluid therapy is required in patients with hypovolaemic shock, and vasopressors should be initiated earlier rather than later if required [58].

Fluid management is particularly challenging in EVD. Excessive fluid may aggravate tissue edema with severe endothelial barrier disruption, while insufficient resuscitation worsens shock-associated hypoperfusion and multiorgan failure [8,14,47,62]. The WHO guidelines provide a structured approach to fluid resuscitation, emphasizing careful monitoring and individualized titration to avoid both under-resuscitation and fluid overload. Advanced intensive care strategies, including vasopressor support, hemodynamic monitoring, renal replacement therapy, and mechanical ventilation, have improved outcomes in high-resource settings [8,27,47]. In the United States and Europe, patients with severe EVD have received mechanical ventilation, renal replacement therapy, invasive monitoring, and vasopressor support, with survival rates above 80% reported in some cohorts [63]. Critical care for patients with EVD is feasible even in resource-limited settings and has been associated with improved survival and reduced hospital stay [63]. Renal dysfunction is common in advanced stages but may be reversible with adequate fluid resuscitation in early stages [63].

Monoclonal antibody therapies such as REGN-EB3 (Inmazeb), a combination of atoltivimab, maftivimab, and odesivimab, and mAb114 (ansuvimab, Ebanga) have substantially modified the therapeutic landscape of disease caused by Ebola virus (*Orthoebolavirus zairense*) [8]. The PALM (Pamoja Tulinde Maisha) randomized controlled trial conducted during the 2018–2020 Kivu outbreak demonstrated the efficacy of these therapies, with 71% survival in patients receiving REGN-EB3 and 66% in those receiving mAb114 [8]. The World Health Organization strongly recommends either REGN-EB3 or mAb114 for patients with confirmed Ebola virus infection [8]. These recommendations apply specifically to disease caused by Ebola virus: the PALM trial enrolled patients infected with this virus, and WHO currently reports no approved therapeutics for other Ebola diseases, including Sudan virus disease and Bundibugyo virus disease, for which candidate products are under development and a core protocol for clinical trials is available [5]. In these diseases, supportive care therefore remains the mainstay of management; the hepatic and endothelial considerations discussed in this review are relevant irrespective of the causative virus, whereas the therapeutic conclusions cannot be extrapolated. Earlier viral control through these antibody therapies may indirectly reduce hepatic injury, endothelial activation, inflammatory amplification, and macrophage-driven tissue damage [8,16,27]. To our knowledge, this hypothesis has not been specifically tested. However, antiviral therapy alone may not fully reverse clinical deterioration once severe endothelial injury, metabolic collapse, and multiorgan dysfunction are established [8,27,62]. In the 2021 Ebola outbreak in Guinea, all eight patients who received Ebola-specific monoclonal antibodies survived, underscoring the life-saving potential of these therapies when administered promptly [63].

Beyond fluid management and antiviral therapy, the management of EVD requires a comprehensive approach to organ support. Coagulopathy and synthetic failure associated with severe hepatic injury necessitate careful management of coagulation parameters, with fresh frozen plasma and platelet transfusions considered in cases of significant bleeding [8,27,47]. Hepatic dysfunction can significantly alter the pharmacokinetics and pharmacodynamics of experimental therapeutics, complicating dosing decisions and increasing the risk of drug toxicity [38,46]. The liver’s role in drug metabolism is often impaired in severe EVD, requiring dose adjustments and careful monitoring of drug levels when available [38,46]. Conversely, investigational and repurposed agents may themselves cause liver injury, which complicates the interpretation of aminotransferase trends during treatment (Table 2). Hepatic and pulmonary macrophage activity in EVD has been demonstrated to involve upregulation of multiple genes and proteins targeted by therapeutics from the field of inflammatory liver disease, offering opportunities for new immunomodulatory strategies [25]. Macrophage-directed therapies, especially those aimed at restoring Kupffer cell homeostasis and reducing the influx of pro-inflammatory monocyte-derived macrophages, represent a promising avenue for mitigating hepatic injury and systemic inflammation [25]. The dynamic changes in the myeloid compartment during Ebola virus pathogenesis suggest that the clinical intervention window for myeloid stabilization in the liver could be broader than that observed in nonhuman primate models, potentially offering a therapeutic opportunity in human disease [25].

The development of targeted therapies that address the underlying mechanisms of hepatic and endothelial injury, rather than merely managing their consequences, represents a promising avenue for improving outcomes in EVD. Several experimental approaches, including modulators of the host inflammatory response, inhibitors of viral entry and replication, and agents that stabilize endothelial barrier function, are currently under investigation and may offer new therapeutic options for patients with severe EVD [49,50,51]. The integration of supportive care with targeted antiviral and immunomodulatory therapies, guided by dynamic biomarker assessment, offers the best opportunity to improve outcomes in this devastating disease. Future research should focus on identifying optimal combinations of supportive care and antiviral therapy, determining the role of immunomodulatory agents, and developing point-of-care tools for dynamic risk stratification and treatment guidance.

## 7. Post-Ebola Syndrome and Long-Term Implications

**Figure 3:**
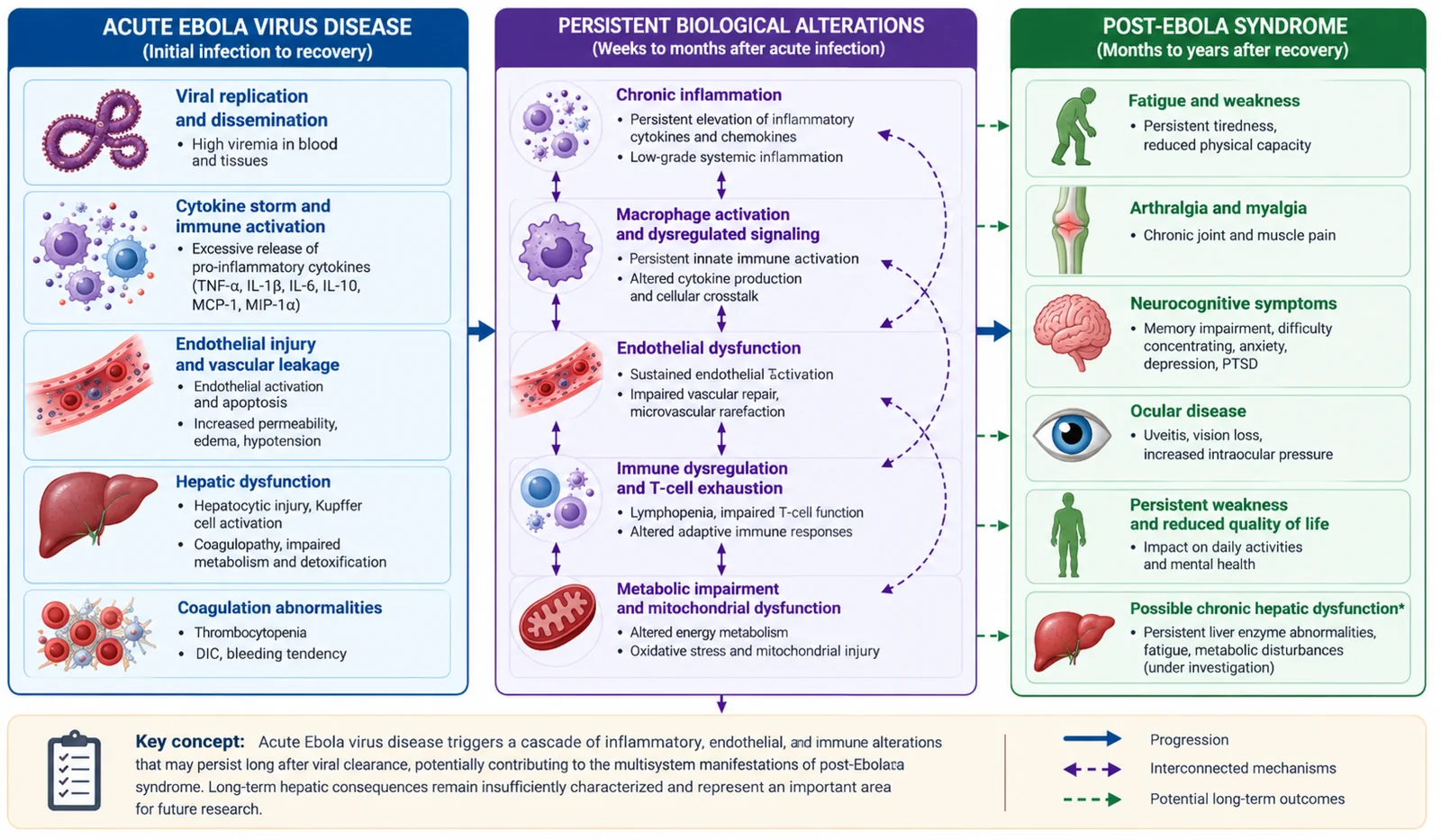
**Proposed mechanisms linking acute Ebola virus disease to post-Ebola syndrome**. Persistent inflammatory activation, endothelial dysfunction, macrophage signaling, and residual tissue injury may contribute to the long-term multisystem sequelae observed among Ebola survivors. Acute EVD triggers a cascade of inflammatory, endothelial, and immune alterations that may persist long after viral clearance, potentially contributing to the multisystem manifestations of post-Ebola syndrome. Long-term hepatic consequences remain insufficiently characterized and represent an important area for future research. The asterisk (*) indicates possible chronic hepatic dysfunction, which remains under investigation and for which evidence is currently limited. Abbreviations: DIC, disseminated intravascular coagulation; EVD, Ebola virus disease; IL, interleukin; MCP-1, monocyte chemoattractant protein-1; MIP-1α, macrophage inflammatory protein-1 alpha; PTSD, post-traumatic stress disorder; TNF-α, tumor necrosis factor alpha.

A substantial proportion of survivors of Ebola virus disease develop persistent clinical manifestations collectively referred to as post-Ebola syndrome (PES), characterized by chronic multisystem symptoms that may persist for months or even years after acute infection [43,44,45,55,56]. As illustrated in Figure 3, the pathophysiology of PES is thought to be driven by persistent inflammatory activation, endothelial dysfunction, macrophage dysregulation, immune exhaustion, and residual tissue injury that extend well beyond the clearance of detectable virus [9,25,27,43,45].

Emerging evidence suggests that the biological consequences of Ebola virus infection may be more persistent than previously appreciated, with inflammatory, endothelial, and immune alterations detectable months to years after recovery [25,27,44,56]. The recognition that PES represents a distinct clinical entity with substantial morbidity has prompted increasing research efforts to characterize its epidemiology, pathophysiology, and long-term health consequences.

Longitudinal cohort studies have documented musculoskeletal, neurological, ophthalmological, constitutional, and systemic symptoms in survivors, with fatigue, arthralgia, visual disturbances, neurocognitive complaints, chronic pain, and persistent weakness among the most frequently reported [43,44,45,55,56]. The clinical presentation of PES is notably heterogeneous. Bond et al. demonstrated that post-Ebola syndrome presents through overlapping symptom clusters rather than isolated organ-specific complications, suggesting a systemic, interconnected pathophysiology rather than discrete end-organ damage [55]. Common symptom clusters include musculoskeletal pain and fatigue; neurocognitive and psychological symptoms, including memory impairment, difficulty concentrating, anxiety, depression, and post-traumatic stress disorder (PTSD); ocular manifestations such as uveitis, vision loss, and increased intraocular pressure; and constitutional symptoms including persistent weakness and reduced quality of life [55]. These symptoms often coexist and may fluctuate over time, complicating both diagnosis and management [55]. The impact on daily activities and mental health is substantial, with many survivors reporting long-term disability and reduced quality of life [43,44,55].

The duration and trajectory of PES symptoms vary considerably among survivors. Diallo et al. described long-term sequelae persisting up to 48 months after infection among Guinean survivors, with a significant proportion experiencing persistent arthralgia, ocular disease, and neurocognitive impairment years after acute infection [43]. The Postebogui cohort study in Guinea documented that post-EVD symptoms can remain long after recovery, with abdominal pain reported in 22% of survivors, justifying calls for regular check-ups of survivors at least 18 months after recovery [43]. More recent investigations identified persistent inflammatory activation among survivors with and without detectable viral RNA persistence, suggesting that immune dysregulation may continue long after viral clearance and may not be solely dependent on viral persistence [56]. Kaweesa et al. documented subclinical organ dysfunction and multisystem sequelae in Sudan Ebola virus (SUDV) survivors two decades after the 2000 outbreak, indicating that some consequences of Ebola virus infection may be extraordinarily long-lasting [44]. Notably, subclinical laboratory deviations centred on hepatic and platelet biology were observed: elevated total bilirubin occurred in 14% of survivors versus 6.7% of controls, while thrombocytopenia or platelet morphological abnormalities were found in 12% versus 3.3% [44]. Similarly, Muwonge et al. found that survivors of Sudan ebolavirus disease continued to experience clinical sequelae two years post-infection, with persistent symptoms affecting multiple organ systems [45]. Of note, both cohorts [44,45] concern Sudan virus disease rather than Ebola virus disease, and their findings cannot be extrapolated to EVD without confirmation; moreover, the reported hepatic and hematological deviations were subclinical, and their clinical relevance remains uncertain. Collectively, these findings underscore the chronic nature of PES and the need for long-term follow-up of survivors.

The mechanisms underlying PES pathogenesis are complex and incompletely understood. Persistent antigenic stimulation and immune-mediated tissue injury are thought to play central roles [56]. Dysregulated cytotoxic T-cell activity and immune checkpoint dysregulation have been observed in Ebola survivors, with elevated levels of pro-inflammatory cytokines and increased CD8+ T-cell activity persisting for months to years after infection [43]. Distinct antibody profiles against Ebola virus have been shown to track with the development of PES, suggesting that persistent antibody-mediated inflammation may contribute to PES manifestations [43]. Autoimmune mechanisms, including molecular mimicry and immune complex deposition into joint tissue, have been proposed as possible contributors to PES pathogenesis, analogous to mechanisms observed in hepatitis arthritis [43]. Furthermore, a systematic review supports persistent inflammation and immune dysregulation in PES, although evidence remains heterogenous and sparse, limiting firm attribution of causality [43]. The potential for viral persistence in immune-privileged sites, with intermittent viral RNA shedding for up to 40 months in some reports, may also contribute to ongoing immune activation and tissue injury [56]. A recent metabolomics study identified alterations in the tricarboxylic acid cycle, amino acid, nucleotide, and short-chain fatty acid metabolism in PES patients, suggesting a metabolic basis for post-infectious sequelae after Ebola virus disease [44].

Compared with acute disease, long-term hepatic consequences remain poorly characterized and represent an important gap in the current understanding of post-Ebola syndrome. Given the extent of hepatic involvement during acute EVD, including direct viral infection of hepatocytes, Kupffer cell activation, inflammatory amplification, and coagulopathy, it is biologically plausible that some survivors experience persistent metabolic, inflammatory, endothelial, or microvascular alterations in the liver [12,13,15,25]. Autopsy studies of EVD consistently report a variable degree of hepatocyte necrosis, accompanied by mild inflammation, mild-to-moderate steatosis, and Kupffer cell hyperplasia [13]. In animal models, the extent of virus replication correlates with elevation of blood liver enzymes (ALT) and progressive deficits in the synthesis of albumin and coagulation factors, which may contribute to several systemic manifestations of EVD [15]. Severe liver impairment is a well-known hallmark of EVD, with hepatic edema, inflammation, and congestion leading to significant hepatomegaly at necropsy [13]. MRI studies have demonstrated progressive hepatocellular dysfunction and hepatic secretory impairment associated with EBOV infection, though the contribution of hepatic pathophysiology to EVD progression is not fully understood [25]. Persistent macrophage activation and low-grade inflammatory signaling, which have been documented in survivors, may contribute to chronic fatigue, constitutional symptoms, metabolic disturbances, or subtle organ dysfunction [9,15,25,27,56]. The potential for chronic hepatic dysfunction in survivors is supported by observations of persistent liver enzyme abnormalities in some cohorts, though systematic longitudinal hepatological data remain limited [43,44,45]. Clinicians are advised to closely monitor ALT, creatinine, and other biomarkers as clinically relevant laboratory parameters to identify survivors at higher risk of developing severe post-acute syndrome upon discharge from Ebola treatment units [44]. The identification of ALT and other biochemical markers as clinically significant predictors of post-acute sequelae underscores the importance of hepatic monitoring in survivor follow-up programs [44]. Possible mechanisms for chronic hepatic sequelae include persistent Kupffer cell activation, low-grade sinusoidal inflammation, microvascular rarefaction, and altered hepatic metabolic regulation [25,27]. However, definitive evidence for clinically significant chronic liver disease in EVD survivors is still lacking, and the relationship between acute hepatic injury severity and long-term outcomes remains to be established [43,44].

The integration of longitudinal liver function testing, imaging, and biomarker analysis into survivor follow-up programs is essential to address these knowledge gaps. Magnetic resonance imaging has shown promise as a non-invasive tool for monitoring hepatic disease induced by Ebola virus, with the potential to detect progressive hepatocellular dysfunction and hepatic secretory impairment [25]. Advanced metabolomic and lipidomic analyses have revealed that EVD survivors exhibit alterations in lipid metabolism, including changes in two-hydroxybutyric acid and 3-hydroxybutyric acid (a ketone body), suggesting excessive fatty acid beta-oxidation and oxidative stress during acute infection, with potential long-term metabolic consequences [44]. The ratios of certain phosphatidylserine lipids correlating with EVD outcome may result from the presence of extracellular vesicles, including exosomes and microvesicles, which could serve as biomarkers of persistent tissue injury [61]. The plasma lipidome may thus provide insights into critical illness and recovery from human Ebola virus disease, offering opportunities for identifying survivors at risk of long-term metabolic dysfunction [44].

Furthermore, understanding the mechanisms that drive persistent hepatic alterations could identify therapeutic targets for the management of chronic post-Ebola manifestations. Given the substantial burden of PES and its impact on quality of life, continued investment in survivor research and clinical care is essential to address the long-term health consequences of Ebola virus disease. Future studies should clarify long-term liver function trajectories, identify possible fibrotic or vascular sequelae, and determine whether the severity of acute hepatic injury influences the later development of chronic post-Ebola manifestations. The emerging evidence of subclinical hepatic and platelet abnormalities in survivors decades after infection argues strongly for survivor-centred long-term care and the integration of hepatic function monitoring into routine follow-up protocols [44]. As the global community continues to confront Ebola virus outbreaks, the long-term health of survivors must remain a priority for both research and clinical practice.

## 8. Discussion

The present review synthesizes the available evidence on hepatic involvement in Ebola virus disease and indicates that liver injury is a prominent and prognostically relevant component of the pathogenic cascade in severe EVD rather than a purely secondary laboratory abnormality. The findings are compatible with a conceptual framework in which EVD is viewed as a multisystem disease with interacting endothelial, inflammatory, and hepatic dysfunction, beyond the classical view of a viral hemorrhagic fever. We use the term “endothelial–hepatic axis” as a heuristic model rather than as a defined clinical or pathological entity: the current evidence is predominantly associative and does not allow us to establish that hepatic injury directly drives endothelial dysfunction, or vice versa. This integrated perspective nevertheless has implications for understanding pathogenesis, identifying prognostic biomarkers, and developing targeted therapeutic strategies.

A key insight emerging from this paper is that hepatic dysfunction in EVD reflects the convergence of multiple interconnected mechanisms rather than a single pathogenic pathway. Direct viral infection of hepatocytes, activation of Kupffer cells, inflammatory amplification, endothelial dysfunction, coagulopathy, and metabolic disturbances all contribute to the progressive liver injury observed in severe disease. The predominance of aspartate aminotransferase over alanine aminotransferase, a frequent finding in severe EVD, is compatible with a multisystem process, potentially reflecting extrahepatic AST release from skeletal muscle, cardiac tissue, and other organs, as well as hemolysis, ischemic injury, and drug effects (Table 2); it therefore cannot, by itself, quantify hepatic damage. This biochemical pattern distinguishes EVD from classical acute viral hepatitis and reinforces the concept that liver test abnormalities should be interpreted as part of a broader systemic syndrome rather than as isolated hepatic findings.

The prognostic significance of hepatic dysfunction is increasingly recognized, with mounting evidence that the degree of liver injury correlates with disease severity and mortality. This evidence is observational: whether hepatic injury is a driver of, or a marker of, systemic severity cannot be resolved with the available data. Elevated aminotransferases, coagulation abnormalities, and metabolic derangements are among the most consistent predictors of adverse outcomes. The identification of these biomarkers has practical implications for clinical management, enabling risk stratification and guiding decisions about the intensity of supportive care. The emergence of machine learning-based prognostic models integrating dynamic biomarker assessment holds promise for further improving risk prediction and facilitating earlier escalation of care.

However, disease severity in EVD is unlikely to depend on any single parameter in isolation, and clinical outcomes probably reflect the cumulative interaction between viral burden, immune responses, endothelial dysfunction, metabolic collapse, and progressive organ injury. A large part of the mechanistic framework discussed in this review rests on non-human primate, murine, and in vitro studies, or is inferred from related conditions, whereas human evidence is mostly descriptive (autopsy series, small clinical cohorts) or associative. To make this explicit, we classified the main statements as established, probable, or hypothetical (see Table 4). Hepatic tropism, hepatocellular necrosis, the association of aminotransferase elevation with mortality, and the occurrence of capillary leak and coagulopathy are established; the molecular mediators of endothelial dysfunction, including glycoprotein–TLR4 signaling, and hepatocellular stress pathways are probable; the reciprocal causal loop between hepatic and endothelial dysfunction, an initiating role of TLR4 activation in patients, a mesenchyme-like transition of hepatocytes, and chronic liver disease in survivors remain hypotheses. Observational associations between liver test abnormalities and mortality are subject to confounding by overall disease severity, viral load, and extrahepatic organ injury, and to reverse causality; they do not, by themselves, indicate that hepatic injury causes clinical deterioration. Prospective studies with serial sampling that combine hepatic (ALT, bilirubin, INR, albumin), muscle (creatine kinase), hemolysis (lactate dehydrogenase, haptoglobin), and endothelial (angiopoietin-2, syndecan-1) markers, with adjustment for confounders and, when feasible, histological or imaging assessment, would help to separate these contributions.

The therapeutic implications of hepatic involvement in EVD are substantial. The recognition that liver dysfunction is embedded within a broader systemic syndrome has shifted the therapeutic paradigm from isolated symptom management toward integrated organ support and hemodynamic optimization. The World Health Organization guidelines for the clinical management of filovirus disease emphasize systematic clinical and laboratory monitoring, oral and intravenous fluid therapy, management of shock, and use of vasopressors as cornerstones of patient-centred supportive care. Monoclonal antibody therapies have substantially modified the treatment landscape of Ebola virus disease, whereas no approved specific therapeutics are currently available for Sudan or Bundibugyo virus disease [5]. Even where antibodies are available, antiviral therapy alone may not fully reverse clinical deterioration once severe endothelial injury, metabolic collapse, and multiorgan dysfunction are established. The dynamic changes in the myeloid compartment during Ebola virus pathogenesis suggest that macrophage-directed therapies, aimed at restoring Kupffer cell homeostasis and reducing the influx of pro-inflammatory monocyte-derived macrophages, may offer a promising therapeutic opportunity. Future research should focus on identifying optimal combinations of supportive care, antiviral therapy, and immunomodulatory agents to improve outcomes in severe EVD.

The long-term consequences of hepatic involvement in EVD remain an important area of uncertainty. While the acute hepatic injury is well documented, the extent to which survivors experience persistent metabolic, inflammatory, endothelial, or microvascular alterations in the liver remains poorly characterized. The emerging evidence of subclinical laboratory deviations centred on hepatic and platelet biology in survivors decades after infection raises important questions about the durability of hepatic recovery and the potential for chronic liver disease in EVD survivors. Longitudinal studies with systematic hepatological assessment are urgently needed to clarify long-term liver function trajectories and to determine whether the severity of acute hepatic injury influences the later development of chronic post-Ebola manifestations. The integration of longitudinal liver function testing, imaging, and biomarker analysis into survivor follow-up programs is essential to address these knowledge gaps and to inform public health policies for the care of survivors.

Several limitations should be acknowledged. The evidence base for hepatic involvement in EVD is largely derived from observational studies, case series, and experimental models, with relatively few prospective clinical studies systematically evaluating liver function parameters. The heterogeneity in study design, case definitions, and outcome measures complicates direct comparisons and limits the generalizability of findings. Furthermore, most studies have focused on the acute phase of infection, with limited longitudinal data on hepatic function in survivors. The lack of standardized protocols for hepatic assessment in EVD and the absence of validated biomarkers for liver injury in this specific context represent additional challenges. The small sample sizes of many studies, particularly those involving human cases, and the inherent difficulties of conducting research in outbreak settings further constrain the evidence base. The reliance on nonhuman primate models, while invaluable for mechanistic studies, may not fully recapitulate all aspects of human disease, particularly the complex interplay between host immune responses and viral pathogenesis. Further limitations of the present review are that the literature search was restricted to PubMed and to English-language publications, that no formal risk-of-bias assessment was performed, and that selection bias therefore cannot be excluded. In addition, the evidence on survivors derives largely from Sudan virus cohorts, and the interpretation of biochemical markers is confounded by extrahepatic sources of aminotransferases (Table 2).

The practical implications of this work extend beyond Ebola virus disease to the broader challenge of understanding organ dysfunction in emerging infectious diseases. The integrated framework presented here provides a template for investigating hepatic involvement in other viral infections characterized by systemic inflammation and multiorgan involvement, such as severe COVID-19, dengue hemorrhagic fever, and other viral hemorrhagic fevers. The principles of integrated organ support, dynamic biomarker assessment, and longitudinal survivor follow-up are applicable to a range of emerging infectious diseases and should be embedded in clinical protocols and research agendas. The experience gained from the Ebola outbreaks in West Africa and the Democratic Republic of the Congo has highlighted the importance of building research capacity in outbreak settings, establishing standardized clinical protocols, and fostering international collaboration to generate robust evidence for clinical practice.

## 9. Conclusions

EVD is a multisystem disorder in which hepatic involvement is a frequent, early, and prognostically relevant feature rather than a mere secondary laboratory finding. Whether hepatic injury acts as a direct driver of disease progression or predominantly as a marker of systemic severity cannot be established from the current, largely associative evidence. The liver is an early and major target of Ebola virus infection, with hepatocytes and Kupffer cells serving as principal sites of viral replication and inflammatory amplification. Hepatic dysfunction arises from the convergence of direct viral cytopathic effects, macrophage activation, endothelial dysfunction, coagulopathy, immune dysregulation, and metabolic disturbances, all of which are tightly interconnected and mutually reinforcing. The clinical spectrum of hepatic involvement ranges from mild transaminase elevation to acute liver failure, and the degree of hepatic injury correlates with disease severity and mortality.

Recognizing the interplay between endothelial and hepatic dysfunction has implications for clinical management. Supportive care remains the cornerstone of treatment, with fluid management, vasopressor support, and organ support guided by dynamic clinical and laboratory assessment. Monoclonal antibody therapies have substantially modified the therapeutic landscape of Ebola virus disease (but not yet of Sudan or Bundibugyo virus disease), yet their integration with comprehensive supportive care remains essential for optimizing outcomes. The long-term health consequences of hepatic involvement in EVD are incompletely understood, and the potential for persistent metabolic, inflammatory, endothelial, or microvascular alterations in survivors warrants systematic investigation.

Future research should focus on elucidating the mechanisms of hepatic injury, developing targeted therapeutic strategies, characterizing long-term outcomes in survivors, and integrating hepatic function monitoring into routine clinical care. The emerging evidence of subclinical hepatic and platelet abnormalities in survivors decades after infection underscores the importance of survivor-centred long-term care. A comprehensive understanding of hepatic involvement in EVD is essential for improving clinical management, developing effective therapeutic strategies, and addressing the long-term health consequences in survivors. As the global community continues to confront Ebola virus outbreaks, the integration of hepatic function assessment into clinical protocols and research agendas represents a critical step toward improving patient outcomes and advancing global health security.

## Figures and Tables

**Table 2:** Sources of aminotransferase and bilirubin elevation in Ebola virus disease and clues to their differentiation. Abbreviations: ALT, alanine aminotransferase; AST, aspartate aminotransferase; CK, creatine kinase; INR, international normalized ratio; LDH, lactate dehydrogenase.

Source	Mechanism	Laboratory and Clinical Clues	References
Direct hepatocellular injury	Viral infection of hepatocytes, Kupffer cell activation, apoptosis/necrosis	Increase of both ALT and AST; ALT may be blunted in fulminant cases; prolonged INR and hypoalbuminemia indicate impaired synthetic function	[1,4,12,36]
Skeletal muscle injury/rhabdomyolysis	Myocyte necrosis with release of AST (and, to a lesser extent, ALT)	Disproportionate AST elevation (AST > ALT); increased CK; myoglobinuria, hyperkalemia, rising creatinine	[1,9,16]
Cardiac and renal injury; mitochondrial dysfunction	Release of AST from cardiac muscle and kidney; systemic mitochondrial stress	AST > ALT; cardiac and renal markers when available	[1,9,16]
Hemolysis	Release of erythrocyte AST and LDH; increased unconjugated bilirubin	Increased LDH and indirect bilirubin; low haptoglobin; anemia; hemolyzed sample	[14]
Ischemic (hypoxic) hepatitis	Shock, hypoperfusion, hypoxia	Abrupt, marked rise of AST, ALT, and LDH with hemodynamic instability; rapid fall after perfusion is restored	[16,48]
Drug-induced liver injury	Investigational/repurposed antivirals, antimalarials, antibiotics, antipyretics	Temporal relationship with drug exposure; improvement after withdrawal; formal causality assessment when feasible	[38,46]
Co-infections and pre-existing liver disease	Malaria, viral hepatitis, other endemic infections; chronic liver disease	Pathogen-specific testing; clinical history	—

**Table 3:** Main clinical and laboratory manifestations associated with hepatic involvement in Ebola virus disease. Abbreviations: ALT, alanine aminotransferase; AST, aspartate aminotransferase; BSEP, bile salt export pump; DIC, disseminated intravascular coagulation; ER, endoplasmic reticulum; IL, interleukin; INR, international normalized ratio; MRP2, multidrug resistance-associated protein 2; PT, prothrombin time; TNF-α, tumor necrosis factor alpha.

Finding	Clinical Significance	Pathophysiological Implication	Key Mechanistic Pathways	References
AST predominance (AST > ALT)	Marker of severe systemic disease with multi-organ involvement	Reflects extensive extrahepatic tissue damage (muscle, heart, kidney) in addition to hepatic injury	AST is localized in mitochondria and cytosolic compartments of liver, cardiac muscle, skeletal muscle, and renal tissue. In EVD, widespread microvascular injury, rhabdomyolysis, and myocardial dysfunction may release AST disproportionately. An AST/ALT ratio > 1 suggests, but does not prove, a substantial extrahepatic contribution (Table 2) and has been associated with severe disease; it is not specific for hepatic injury.	[1,6,9,16,33,35,37]
Elevated ALT	Indicator of hepatocellular injury	Correlates with direct viral cytopathic effect and inflammatory necrosis	ALT is predominantly cytosolic and more liver-specific than AST. Its elevation reflects is thought to reflect hepatocyte membrane disruption due to viral replication, ER stress, and caspase-mediated apoptosis. The magnitude of ALT rise parallels the degree of parenchymal inflammation and necrosis, though it may be blunted in fulminant cases due to massive hepatocyte depletion.	[1,4,12,36]
Hyperbilirubinemia	Sign of advanced disease and hepatic excretory dysfunction	Reflects impaired bilirubin conjugation and canalicular transport	Elevated total and direct bilirubin is thought to result from dysfunction of sinusoidal and canalicular transporters (e.g., MRP2, BSEP) due to cytokine-mediated cholestasis and direct viral injury to biliary epithelia. It is associated with prolonged disease course, higher viral loads, and increased mortality. Hemolysis may contribute an unconjugated (indirect) component (Table 2).	[1,12,14]
Thrombocytopenia	Common in severe EVD; correlates with hemorrhagic risk	Reflects consumptive coagulopathy, endothelial activation, and marrow suppression	Platelet count drops due to consumption in microthrombi, direct viral infection of megakaryocytes, and immune-mediated clearance. Endothelial injury exposes subendothelial collagen, activating platelets and the coagulation cascade. Severe thrombocytopenia (< 50 × 10^9^/L) is a strong predictor of bleeding complications and fatal outcome.	[9,16,26,48]
Prolonged INR/PT	Marker associated with poor prognosis	Suggests hepatic synthetic dysfunction, often with a consumptive (DIC-like) component, and impaired production of coagulation factors	The liver synthesizes all vitamin K-dependent clotting factors (II, VII, IX, X). Prolonged prothrombin time (PT) and elevated international normalized ratio (INR) reflect hepatocellular loss and functional impairment. In EVD, this is compounded by accelerated consumption due to DIC, leading to a vicious cycle of bleeding and organ failure.	[9,16,26,48]
Hypoalbuminemia	Marker of critical illness and poor nutritional/reserve status	Reflects reduced hepatic synthesis compounded by capillary leakage and albumin redistribution	Albumin is exclusively synthesized in the liver. Low levels result from decreased hepatocellular mass, downregulation of albumin gene transcription by inflammatory cytokines (IL-6, TNF-α), and increased vascular permeability with extravasation into interstitial spaces. Hypoalbuminemia is associated with edema, ascites, and worse survival.	[9,16,48]

**Table 4:** Strength of evidence supporting key statements of the endothelial–hepatic framework. Established: consistent findings in human data (autopsy, clinical cohorts) or replicated across models with human corroboration. Probable: consistent experimental (non-human primate, animal, in vitro) evidence with limited or indirect human data. Hypothetical: inferred from related conditions, from single in vitro studies, or from conceptual synthesis, without direct supporting data. Abbreviations: EBOV, Ebola virus; GP, glycoprotein; NHP, non-human primate; SUDV, Sudan virus; TGF-β, transforming growth factor beta; TLR4, Toll-like receptor 4.

Statement	Main Supporting Evidence	Level	Comment on Causality/Limitations
The liver is an early and major target of EBOV; hepatocytes and Kupffer cells are infected	Human autopsy and tissue studies [13]; NHP and humanized mouse studies [15,28]	Established	Direct evidence of tropism; contribution of infection to organ dysfunction is inferred
Elevated AST/ALT at presentation are associated with severe disease and death	Observational human cohorts and outbreak series [33,35,37]	Established (association)	Not specific for hepatic injury; confounded by overall severity and extrahepatic sources (Table 2); causality not tested
Impaired hepatic synthetic function contributes to coagulopathy	Clinical laboratory findings [16,48]; NHP [15]	Probable	DIC-like consumption is a concurrent contributor; relative weights unknown
Vascular leakage results from endothelial activation and barrier dysfunction driven by GP and cytokines	Clinical descriptions [16]; in vitro and animal models [31,60]	Probable	Mostly experimental; infection-independent mechanisms predominate; endothelial infection is a late event
Shed GP activates TLR4 signaling	In vitro and animal studies [19,20,21]	Probable	No direct human evidence
TLR4 activation by shed GP is a key initiating event of inflammation in patients	Extrapolation from experimental data	Hypothetical	Not demonstrated in humans
Hepatocyte ER stress, mitochondrial dysfunction, and apoptosis contribute to hepatic injury	In vitro (including iPSC-derived hepatocytes) and NHP studies [17,18,28]	Probable	Limited evidence in human liver tissue
Viral modulation of TGF-β induces a mesenchyme-like transition and fibrosis in hepatocytes	Single in vitro study [17]	Hypothetical	No in vivo or human confirmation
Hepatic injury drives progression to multiorgan failure (rather than marking systemic severity)	Association only [33,35,37]	Hypothetical	Confounding and reverse causality cannot be excluded
Bidirectional amplification between hepatic and endothelial dysfunction (“endothelial–hepatic axis”)	Conceptual synthesis	Hypothetical	Not directly tested
Chronic liver disease occurs in survivors	Subclinical laboratory deviations in SUDV cohorts [44,45]; PostEboGui cohort [43]; no histology or imaging	Hypothetical	Insufficient evidence for chronic liver disease; mostly SUDV

## Data Availability

No new data were created or analyzed in this study. Data sharing is not applicable to this article.

## References

[1] van Leeuwen L.P., de Jong W., Doornekamp L., van Gorp E.C., Wismans P.J., Goeijenbier M. (2022). Exotic viral hepatitis: A review on epidemiology, pathogenesis, and treatment. J. Hepatol..

[2] Feldmann H., Sprecher A., Geisbert T.W. (2020). Ebola. N. Engl. J. Med..

[3] World Health Organization (WHO) 2015 WHO Strategic Response Plan: West Africa Ebola Outbreak. https://iris.who.int/items/bf81c9f8-3d21-4cae-8c26-e6cf6f0cb80c.

[4] Coltart C.E., Lindsey B., Ghinai I., Johnson A.M., Heymann D.L. (2017). The Ebola outbreak, 2013–2016: Old lessons for new epidemics. Philos. Trans. R. Soc. B Biol. Sci..

[5] World Health Organization (WHO) Ebola Disease Caused by Bundibugyo Virus—Democratic Republic of the Congo. https://www.who.int/emergencies/disease-outbreak-news/item/2026-DON618.

[6] Barry A., Ahuka-Mundeke S., Ahmed Y.A., Allarangar Y., Anoko J., Archer B.N., Abedi A.A., Bagaria J., Belizaire M.R.D., Bhatia S. (2018). Outbreak of Ebola virus disease in the Democratic Republic of the Congo, April–May, 2018: An epidemiological study. Lancet.

[7] World Health Organization (WHO) Uganda Declares End of Ebola Disease Outbreak. https://www.afro.who.int/countries/uganda/news/uganda-declares-end-ebola-disease-outbreak.

[8] Mulangu S., Dodd L.E., Davey R.T., Tshiani Mbaya O., Proschan M., Mukadi D., Lusakibanza Manzo M., Nzolo D., Tshomba Oloma A., Ibanda A. (2019). A randomized, controlled trial of Ebola virus disease therapeutics. N. Engl. J. Med..

[9] Baseler L., Chertow D.S., Johnson K.M., Feldmann H., Morens D.M. (2017). The pathogenesis of Ebola virus disease. Annu. Rev. Pathol. Mech. Dis..

[10] Muñoz-Fontela C., McElroy A.K., Mühlberger E., Hensley L.L., Towner J.S. (2017). Ebola virus disease in humans: Pathophysiology and immunity. Marburg- and Ebolaviruses: From Ecosystems to Molecules.

[11] Bwaka M.A., Bonnet M.J., Calain P., Colebunders R., De Roo A., Guimard Y., Katwiki K.R., Kibadi K., Kipasa M.A., Kuvula K.J. (1999). Ebola hemorrhagic fever in Kikwit, Democratic Republic of the Congo: Clinical observations in 103 patients. J. Infect. Dis..

[12] Cheng Y., Zheng X. (2025). Characteristics and mechanisms of liver injury caused by emerging infectious diseases. Front. Immunol..

[13] Martines R.B., Ng D.L., Greer P.W., Rollin P.E., Zaki S.R. (2015). Tissue and cellular tropism, pathology and pathogenesis of Ebola and Marburg viruses. J. Pathol..

[14] Fausther-Bovendo H., Qiu X., Babuadze G.G., Azizi H., Pedersen J., Wong G., Kobinger G.P. (2022). Transient liver damage and hemolysis are associated with an inhibition of ebola virus glycoprotein-specific antibody response and lymphopenia. J. Infect. Dis..

[15] Spengler J.R., Saturday G., Lavender K.J., Martellaro C., Keck J.G., Nichol S.T., Spiropoulou C.F., Feldmann H., Prescott J. (2018). Severity of disease in humanized mice infected with Ebola virus or Reston virus is associated with magnitude of early viral replication in liver. J. Infect. Dis..

[16] Wolf T., Kann G., Becker S., Stephan C., Brodt H.R., de Leuw P., Grünewald T., Vogl T., Kempf V.A., Keppler O.T. (2015). Severe Ebola virus disease with vascular leakage and multiorgan failure: Treatment of a patient in intensive care. Lancet.

[17] Kindrachuk J., Wahl-Jensen V., Safronetz D., Trost B., Hoenen T., Arsenault R., Feldmann F., Traynor D., Postnikova E., Kusalik A. (2014). Ebola virus modulates transforming growth factor *β* signaling and cellular markers of mesenchyme-like transition in hepatocytes. J. Virol..

[18] Scoon W.A., Mancio-Silva L., Suder E.L., Villacorta-Martin C., Lindstrom-Vautrin J., Bernbaum J.G., Mazur S., Johnson R.F., Olejnik J., Flores E.Y. (2022). Ebola virus infection induces a delayed type I IFN response in bystander cells and the shutdown of key liver genes in human iPSC-derived hepatocytes. Stem Cell Rep..

[19] Okumura A., Pitha P.M., Yoshimura A., Harty R.N. (2010). Interaction between Ebola virus glycoprotein and host toll-like receptor 4 leads to induction of proinflammatory cytokines and SOCS1. J. Virol..

[20] Lai C.Y., Strange D.P., Wong T.A.S., Lehrer A.T., Verma S. (2017). Ebola virus glycoprotein induces an innate immune response in vivo via TLR4. Front. Microbiol..

[21] Scherm M.J., Gangloff M., Gay N.J. (2022). Activation of Toll-like receptor 4 by Ebola virus-shed glycoprotein is direct and requires the internal fusion loop but not glycosylation. Cell Rep..

[22] Wanninger T.G., Millian D.E., Saldarriaga O.A., Maruyama J., Saito T., Reyna R.A., Taniguchi S., Arroyave E., Connolly M.E., Stevenson H.L. (2022). Macrophage infection, activation, and histopathological findings in ebolavirus infection. Front. Cell. Infect. Microbiol..

[23] Bray M., Geisbert T.W. (2005). Ebola virus: The role of macrophages and dendritic cells in the pathogenesis of Ebola hemorrhagic fever. Int. J. Biochem. Cell Biol..

[24] Stein S.R., Platt A.P., Teague H.L., Anthony S.M., Reeder R.J., Cooper K., Byrum R., Drawbaugh D.J., Liu D.X., Burdette T.L. (2023). Clinical and Immunologic Correlates of Vasodilatory Shock Among Ebola Virus–Infected Nonhuman Primates in a Critical Care Model. J. Infect. Dis..

[25] Tseng A.E., Carossino M., Gertje H.P., O’Connell A.K., Gummuluru S., Kolachalama V.B., Balasuriya U.B., Connor J.H., Bennett R.S., Liu D.X. (2023). Hepatic proinflammatory myeloid phenotypes are a hallmark of Ebola virus Kikwit pathogenesis in rhesus monkeys. Vet. Pathol..

[26] Banerjee G., Shokeen K., Chakraborty N., Agarwal S., Mitra A., Kumar S., Banerjee P. (2021). Modulation of immune response in Ebola virus disease. Curr. Opin. Pharmacol..

[27] Kotliar D., Lin A.E., Logue J., Hughes T.K., Khoury N.M., Raju S.S., Wadsworth M.H., Chen H., Kurtz J.R., Dighero-Kemp B. (2020). Single-cell profiling of Ebola virus disease in vivo reveals viral and host dynamics. Cell.

[28] Jankeel A., Menicucci A.R., Woolsey C., Fenton K.A., Mendoza N., Versteeg K., Cross R.W., Geisbert T.W., Messaoudi I. (2020). Early transcriptional changes within liver, adrenal gland, and lymphoid tissues significantly contribute to ebola virus pathogenesis in cynomolgus macaques. J. Virol..

[29] McElroy A.K., Erickson B.R., Flietstra T.D., Rollin P.E., Nichol S.T., Towner J.S., Spiropoulou C.F. (2014). Ebola hemorrhagic fever: Novel biomarker correlates of clinical outcome. J. Infect. Dis..

[30] Wauquier N., Becquart P., Padilla C., Baize S., Leroy E.M. (2010). Human fatal zaire ebola virus infection is associated with an aberrant innate immunity and with massive lymphocyte apoptosis. PLoS Negl. Trop. Dis..

[31] Wahl-Jensen V.M., Afanasieva T.A., Seebach J., Ströher U., Feldmann H., Schnittler H.J. (2005). Effects of Ebola virus glycoproteins on endothelial cell activation and barrier function. J. Virol..

[32] Nakayama E., Saijo M. (2013). Animal models for Ebola and Marburg virus infections. Front. Microbiol..

[33] Schieffelin J.S., Shaffer J.G., Goba A., Gbakie M., Gire S.K., Colubri A., Sealfon R.S., Kanneh L., Moigboi A., Momoh M. (2014). Clinical illness and outcomes in patients with Ebola in Sierra Leone. N. Engl. J. Med..

[34] Lado M., Walker N.F., Baker P., Haroon S., Brown C.S., Youkee D., Studd N., Kessete Q., Maini R., Boyles T. (2015). Clinical features of patients isolated for suspected Ebola virus disease at Connaught Hospital, Freetown, Sierra Leone: A retrospective cohort study. Lancet Infect. Dis..

[35] Bah E.I., Lamah M.C., Fletcher T., Jacob S.T., Brett-Major D.M., Sall A.A., Shindo N., Fischer W.A., Lamontagne F., Saliou S.M. (2015). Clinical presentation of patients with Ebola virus disease in Conakry, Guinea. N. Engl. J. Med..

[36] Qin E., Bi J., Zhao M., Wang Y., Guo T., Yan T., Li Z., Sun J., Zhang J., Chen S. (2015). Clinical features of patients with Ebola virus disease in Sierra Leone. Clin. Infect. Dis..

[37] Hunt L., Gupta-Wright A., Simms V., Tamba F., Knott V., Tamba K., Heisenberg-Mansaray S., Tamba E., Conteh S., Smith T. (2015). Clinical presentation, biochemical, and haematological parameters and their association with outcome in patients with Ebola virus disease: An observational cohort study. Lancet Infect. Dis..

[38] Sissoko D., Duraffour S., Kerber R., Kolie J.S., Beavogui A.H., Camara A.M., Colin G., Rieger T., Oestereich L., Pályi B. (2017). Persistence and clearance of Ebola virus RNA from seminal fluid of Ebola virus disease survivors: A longitudinal analysis and modelling study. Lancet Glob. Health.

[39] Chertow D.S., Kleine C., Edwards J.K., Scaini R., Giuliani R., Sprecher A. (2014). Ebola virus disease in West Africa—clinical manifestations and management. N. Engl. J. Med..

[40] Faye O., Boëlle P.Y., Heleze E., Faye O., Loucoubar C., Magassouba N., Soropogui B., Keita S., Gakou T., Bah E.H.I. (2015). Chains of transmission and control of Ebola virus disease in Conakry, Guinea, in 2014: An observational study. Lancet Infect. Dis..

[41] Kortepeter M.G., Bausch D.G., Bray M. (2011). Basic clinical and laboratory features of filoviral hemorrhagic fever. J. Infect. Dis..

[42] Bausch D.G., Towner J.S., Dowell S.F., Kaducu F., Lukwiya M., Sanchez A., Nichol S.T., Ksiazek T.G., Rollin P.E. (2007). Assessment of the risk of Ebola virus transmission from bodily fluids and fomites. J. Infect. Dis..

[43] Diallo M.S.K., Toure A., Sow M.S., Kpamou C., Keita A.K., Taverne B., Peeters M., Msellati P., Barry T.A., Etard J.F. (2021). Understanding long-term evolution and predictors of sequelae of Ebola virus disease survivors in Guinea: A 48-month prospective, longitudinal cohort study (PostEboGui). Clin. Infect. Dis..

[44] Kaweesa R.E., Katende J.S., Odoch G., Ntabadde A.D., Wayesu R.R., Mukisa D., Ejou P., Team F., Kaleebu P., Serwanga J. (2025). Two Decades Later: Long-Term Multisystem Sequelae and Subclinical Organ Dysfunction in Sudan Ebola Virus (SUDV) Survivors of the 2000 Outbreak. Viruses.

[45] Muwonge H., Nasimiyu C., Bakamutumaho B., Elyanu P., Joloba M.L., Situma S., Schieffelin J., Gunn B., Bai S., Breiman R.F. (2025). Long-term clinical sequelae among Sudan ebolavirus disease survivors 2 years post-infection: A matched cohort study. BMC Med..

[46] Dunning J., Kennedy S.B., Antierens A., Whitehead J., Ciglenecki I., Carson G., Kanapathipillai R., Castle L., Howell-Jones R., Pardinaz-Solis R. (2016). Experimental treatment of Ebola virus disease with brincidofovir. PLoS ONE.

[47] Sissoko D., Folkesson E., Abdoul M., Beavogui A.H., Gunther S., Shepherd S., Danel C., Mentre F., Anglaret X., Malvy D. (2015). Favipiravir in patients with Ebola virus disease: Early results of the JIKI trial in Guinea. Proceedings of the Conference on Retroviruses and Opportunistic Infections.

[48] Leligdowicz A., Fischer W.A., Uyeki T.M., Fletcher T.E., Adhikari N.K., Portella G., Lamontagne F., Clement C., Jacob S.T., Rubinson L. (2016). Ebola virus disease and critical illness. Crit. Care.

[49] Sweiti H., Ekwunife O., Jaschinski T., Lhachimi S.K. (2017). Repurposed therapeutic agents targeting the Ebola virus: A systematic review. Curr. Ther. Res..

[50] Cardile A.P., Mayers D.L., Bavari S. (2014). Current status of chemically synthesized inhibitors of Ebola virus. Recent Pat. Anti-Infect. Drug Discov..

[51] Cross R.W., Mire C.E., Feldmann H., Geisbert T.W. (2018). Post-exposure treatments for Ebola and Marburg virus infections. Nat. Rev. Drug Discov..

[52] Reynolds P., Marzi A. (2017). Ebola and Marburg virus vaccines. Virus Genes.

[53] Jacob S.T., Crozier I., Fischer W.A., Hewlett A., Kraft C.S., Vega M.A.d.L., Soka M.J., Wahl V., Griffiths A., Bollinger L. (2020). Ebola virus disease. Nat. Rev. Dis. Prim..

[54] Geisbert T.W., Hensley L.E., Jahrling P.B., Larsen T., Geisbert J.B., Paragas J., Young H.A., Fredeking T.M., Rote W.E., Vlasuk G.P. (2003). Treatment of Ebola virus infection with a recombinant inhibitor of factor VIIa/tissue factor: A study in rhesus monkeys. Lancet.

[55] Bond N.G., Grant D.S., Himmelfarb S.T., Engel E.J., Al-Hasan F., Gbakie M., Kamara F., Kanneh L., Mustapha I., Okoli A. (2021). Post-Ebola syndrome presents with multiple overlapping symptom clusters: Evidence from an ongoing cohort study in Eastern Sierra Leone. Clin. Infect. Dis..

[56] Fallah M.P., Van Ryn C., Moses J.S., Badio M., Fayiah T., Johnson K., Gayedyu-Dennis D., Eghrari A.O., Weiser S.D., Porco T.C. (2025). Associations of inflammatory markers with post-acute clinical findings among survivors of Ebola virus disease with and without viral RNA shedding in the semen in Liberia: A nested case–control study. Lancet Microbe.

[57] Sui S., Zhang K., Shen X., Chuai X., Chiu S. (2026). Liver injury during Ebola virus infections: Characteristics and molecular mechanisms. Virol. Sin..

[58] World Health Organization (WHO) WHO Guidelines for the Clinical Management of Filovirus Disease. https://www.who.int/publications/i/item/B09774.

[59] Kebela J.K., Kimpanga P., Kyloka J.N., Musema G., Mabela R., Bigrimana R., Mangapi O., Barhayiga B., Mwokozi E.B., Ntumba S. (2025). Bayesian predictive model of Ebola fatality: Tenth Ebola epidemic in the Democratic Republic of the Congo. J. Public Health Afr..

[60] Moni B.M., Sakurai Y., Yasuda J. (2022). Ebola virus GP activates endothelial cells via host cytoskeletal signaling factors. Viruses.

[61] Vucetic A., Lafleur A., Côté M., Kobasa D., Chan M., Alvarez F., Piccirillo C., Dong G., Olivier M. (2023). Extracellular vesicle storm during the course of Ebola virus infection in primates. Front. Cell. Infect. Microbiol..

[62] Marzi A., Feldmann H. (2014). Ebola virus vaccines: An overview of current approaches. Expert Rev. Vaccines.

[63] Bearnot C.J., Mbong E.N., Muhayangabo R.F., Laghari R., Butler K., Gainey M., Perera S.M., Michelow I.C., Tang O.Y., Levine A.C. (2024). Derivation and Internal Validation of a Mortality Prognostication Machine Learning Model in Ebola Virus Disease Based on Iterative Point-of-Care Biomarkers. Proceedings of the Open Forum Infectious Diseases.

